# Two novel types of hexokinases in the moss *Physcomitrella patens*

**DOI:** 10.1186/1471-2229-11-32

**Published:** 2011-02-14

**Authors:** Anders Nilsson, Tina Olsson, Mikael Ulfstedt, Mattias Thelander, Hans Ronne

**Affiliations:** 1 Department of Microbiology, Swedish University of Agricultural Sciences, Box 7025, SE-750 07 Uppsala, Sweden; 2 Department of Plant Biology and Forest Genetics, Swedish University of Agricultural Sciences, Box 7080, SE-750 07 Uppsala, Sweden

## Abstract

**Background:**

Hexokinase catalyzes the phosphorylation of glucose and fructose, but it is also involved in sugar sensing in both fungi and plants. We have previously described two types of hexokinases in the moss *Physcomitrella*. Type A, exemplified by PpHxk1, the major hexokinase in *Physcomitrella*, is a soluble protein that localizes to the chloroplast stroma. Type B, exemplified by PpHxk2, has an N-terminal membrane anchor. Both types are found also in vascular plants, and localize to the chloroplast stroma and mitochondrial membranes, respectively.

**Results:**

We have now characterized all 11 hexokinase encoding genes in *Physcomitrella*. Based on their N-terminal sequences and intracellular localizations, three of the encoded proteins are type A hexokinases and four are type B hexokinases. One of the type B hexokinases has a splice variant without a membrane anchor, that localizes to the cytosol and the nucleus. However, we also found two new types of hexokinases with no obvious orthologs in vascular plants. Type C, encoded by a single gene, has neither transit peptide nor membrane anchor, and is found in the cytosol and in the nucleus. Type D hexokinases, encoded by three genes, have membrane anchors and localize to mitochondrial membranes, but their sequences differ from those of the type B hexokinases. Interestingly, all moss hexokinases are more similar to each other in overall sequence than to hexokinases from other plants, even though characteristic sequence motifs such as the membrane anchor of the type B hexokinases are highly conserved between moss and vascular plants, indicating a common origin for hexokinases of the same type.

**Conclusions:**

We conclude that the hexokinase gene family is more diverse in *Physcomitrella*, encoding two additional types of hexokinases that are absent in vascular plants. In particular, the presence of a cytosolic and nuclear hexokinase (type C) sets *Physcomitrella *apart from vascular plants, and instead resembles yeast, where all hexokinases localize to the cytosol. The fact that all moss hexokinases are more similar to each other than to hexokinases from vascular plants, even though both type A and type B hexokinases are present in all plants, further suggests that the hexokinase gene family in *Physcomitrella *has undergone concerted evolution.

## Background

Hexokinases catalyze the first step in hexose metabolism, the phosphorylation of glucose and fructose. Hexokinases that show a higher specificity for glucose than for fructose are sometimes called glucokinases. The yeast *Saccharomyces *thus has a glucokinase, ScGlk1, and two dual specificity hexokinases, ScHxk1 and ScHxk2. The eukaryotic hexokinases are all related to each other, but are unrelated to prokaryotic glucokinases and hexokinases. Plants also have a fructokinase which is unrelated to the hexokinases [1-3].

Hexokinases are found in several different intracellular locations. The three yeast hexokinases are cytosolic, but ScHxk2 can also enter the nucleus [4]. Animal type I and II hexokinases have hydrophobic N-termini that target them to the outer mitochondrial membrane, whereas type III and IV hexokinases are cytosolic, but the latter can also enter the nucleus [3]. We have previously described two types of plant hexokinases [5]. Type A is exemplified by the *Physcomitrella *hexokinase PpHxk1, a soluble protein with a transit peptide [6] that localizes to the chloroplast stroma. Type B hexokinases exemplified by PpHxk2, have N-terminal membrane anchors. Both types are present also in vascular plants, where they localize to the chloroplast stroma and to the outer mitochondrial membrane, respectively [7-14].

In addition to their metabolic roles, eukaryotic hexokinases have also been implicated in signal transduction. Mitochondria-associated hexokinases have thus been shown to negatively affect programmed cell death in both animals and plants, by preventing the release of cytochrome c from mitochondria [14-17]. It should be noted, however, that this does not prove that a signal is transmitted by hexokinase, which could have a constitutive inhibitory effect on cytochrome c release. A more direct role for hexokinases in signal transduction is suggested by studies of the response to glucose in several organisms. Thus, early work in yeast showed that ScHxk2 is required for glucose repression [18,19], but the molecular mechanism has resisted analysis for more than 30 years [20-23].

An important question is where the enzyme exerts its signaling function. Early work in yeast focused on the cytosol, since the yeast hexokinases are cytosolic. However, further studies have shown that ScHxk2 also can translocate into the nucleus, where it forms a complex with the Mig1 repressor [4,24]. Similarly, evidence from *Arabidopsis *[25] and rice [26,27] suggest that plant type B hexokinases may enter the nucleus and participate in gene regulation.

The moss *Physcomitrella patens *is unique among plants in that gene targeting by homologous recombination works in it with frequencies comparable to yeast [28]. This has made *Physcomitrella *a powerful model system for studies of plant gene function [29,30]. The recent sequencing of the *Physcomitrella *genome has further strengthened it as a model plant [31]. We have previously characterized the *Physcomitrella *hexokinase PpHxk1, which by gene targeting was shown to account for 80% of the glucose phosphorylating activity in protonemal tissue [5]. Further studies of a PpHxk1 knockout mutant revealed a number of interesting phenotypes, but no conclusive evidence was obtained as to the possible role of this hexokinase in signaling [32]. Part of the problem is that *Physcomitrella *like other plants possesses several hexokinases, which makes it difficult to draw conclusions about gene function from the knockout of a single gene.

We here report the characterization of all eleven genes encoding putative hexokinase proteins in the *Physcomitrella *genome. Seven of the genes predict proteins that clearly belong to the previously described types A and B [5]. However, the remaining four genes encode two novel types of hexokinases, which we call C and D. The type C hexokinase PpHxk4 is a soluble protein which lacks both organelle targeting peptide and membrane anchor. The three type D hexokinases PpHxk9, PpHxk10 and PpHxk11 resemble the type B hexokinases in that they possess hydrophobic membrane anchors, but differ in sequence from the latter. The type D hexokinases also have a similar localization as the type B hexokinases, being found in the outer mitochondrial membrane, and to some extent in the chloroplast envelope.

## Methods

### Plant material and growth conditions

The growth conditions used were growth at 25°C under constant light in a Sanyo MLR-350 light chamber with irradiation from the sides. Light was supplied from fluorescent tubes (FL40SS W/37, Toshiba) at 30 μmol m^-2 ^s^-1^. Subculturing of *Physcomitrella patens *protonemal tissue was done on cellophane overlaid 0.8% agar plates containing BCD media (1 mM MgSO_4_, 1.85 mM KH_2_PO_4_, 10 mM KNO_3_, 45 μM FeSO_4_, 1 mM CaCl_2_, and trace elements [33]), supplemented with 5 mM ammonium tartrate.

### Cloning of hexokinase cDNAs and genomic sequences

In the same degenerative polymerase chain reaction (PCR) where we isolated *PpHXK1 *we also found several other hexokinase encoding sequences [5]. From these, we could design primers to amplify full length cDNAs and genes of *PpHXK2 *and *PpHXK3 *(Additional files 1 and 2: Tables S1 and S2). The sequences of *PpHXK1 *and *PpHXK2 *were then used to search the PHYSCObase EST data base [34] for more hexokinase sequences. Based on the sequences found, primers were designed to amplify the *PpHXK4 *gene and cDNA and the *PpHXK5 *gene. Several of the partially sequenced EST clones identified in the PHYSCObase were also ordered from the RIKEN bioresource center and fully sequenced (Additional file 1: Table S1). When the sequence of the *Physcomitrella patens *genome became available [31], we searched it for additional hexokinase encoding sequences. This revealed six more putative hexokinase genes: *PpHXK6*-*PpHXK11*. Primers were designed to amplify genes and cDNAs of these hexokinases (Additional files 1 and 2: Tables S1 and S2).

### GFP fusions and localization studies

In our localization studies we used the Green Fluorescent Protein (GFP) from the vector psmRS-GFP, a pUC118 based plasmid with the 35S promoter in front of a soluble modified red shifted GFP followed by the *NOS1 *terminator [35]. Primers ending with *Bam*HI or *Bgl*II sites were designed to facilitate sticky end ligation of PCR products into the *Bam*HI site between the 35S promoter and the rsGFP coding region (Additional file 2: Table S2). GFP fusions were made for all eleven *Physcomitrella *hexokinases. For PpHXK2, 3, 4, and 7 the full length cDNAs were fused in frame to GFP, but for PpHXK5, 8, 9, 10 and 11 partial cDNAs were used since no full length cDNAs were available. No cDNA was available for PpHXK6, so the first exon amplified from the genomic DNA was used to construct a GFP fusion in that case. For all hexokinases two different versions of the hexokinase-GFP fusions were made: one containing the N-terminal membrane anchor or chloroplast transit peptide and one in which the membrane anchor or chloroplast transit peptide had been deleted (Additional file 3: Table S3). For PpHxk10 a hexokinase-GFP fusion was also made where the membrane anchor of PpHxk10 was fused directly to GFP.

GFP fusion constructs were transiently expressed in wild type protoplasts after PEG-mediated transformation [36]. The transformed protoplasts were analyzed after one to two days of incubation in the dark in a Zeiss Axioskop 2 mot fluorescence light microscope equipped with either a HR or MRm AxioCam camera from Zeiss. The GFP signal was detected using a FITC filter (excitation 480 nm, emission 535 nm, dichronic beamsplitter 505 nm) while chloroplast autofluorescence was detected using a TRITC filter (excitation 535 nm, emission 620 nm, dichronic beamsplitter 565 nm). The mitochondria specific dye MitoTracker^® ^Orange was detected with Zeiss filter set number 20 (excitation 546/12 nm, emission 575-640 nm, dichronic beamsplitter 560 nm). The nucleic acid stain 4',6-diamidino-2-phenylindole dihydrochloride (DAPI) was used to visualize the nucleus and detected using a DAPI/Hoechst filter (excitation 360 nm, emission 460 nm, dichronic beamsplitter 400 nm).

### Yeast complementation experiments

A yeast strains with triple knockouts of the *HXK1*, *HXK2 *and *GLK1 *genes in the W303-1A background [37] was kindly provided by Stefan Hohmann [20]. Hexokinase-encoding cDNA sequences from *Physcomitrella *were cloned into the high copy number 2 μm *URA3 *shuttle vector pFL61 [38], which expresses inserts in yeast from the constitutive *PGK *promoter (Additional files 2 and 3: Tables S2 and S3). Transformants were selected on synthetic media lacking uracil, with 2% galactose as carbon source in order to permit hexokinase deficient strains to grow. Colonies were picked to synthetic galactose plates lacking uracil, and the resulting grids were replicated to synthetic media lacking uracil and containing different carbon sources. Growth was scored after 6 days at 30°C.

### Sequence analysis

The Vector NTI software package with ContigExpress (Invitrogen) was used for sequence editing, sequence analysis and building of contigs. The sequence of PpHxk1 differs in one position (leucine-55) from the published sequence [5] due to a sequence error that has now been corrected in GenBank. For the tree-building, we used the Neighbour-Joining method [39] as previously described [40].

## Results

### The *Physcomitrella *genome encodes eleven putative hexokinases

**Figure 1:**
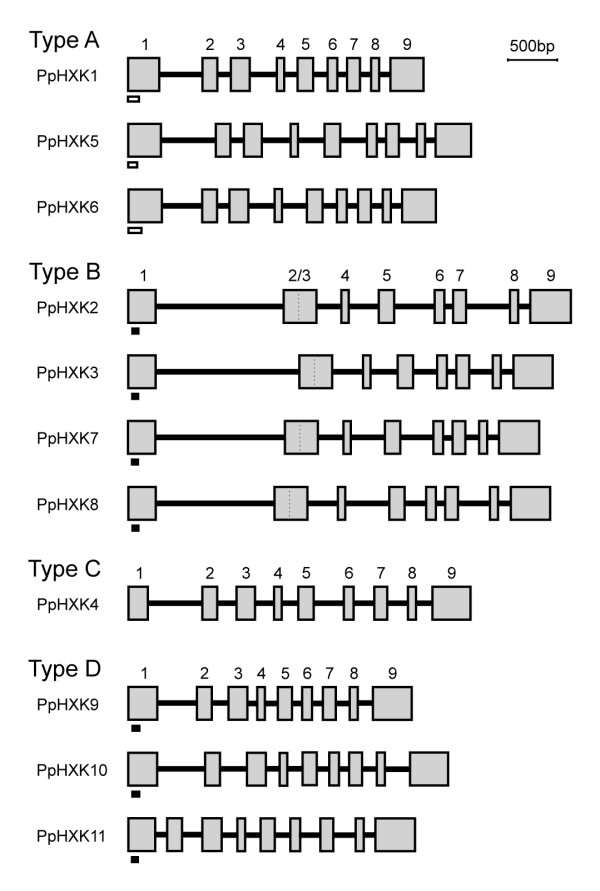
**Overview of the hexokinase genes in *Physcomitrella***. Exons are shown as gray boxes and introns as solid black lines. The predicted exon/intron organization is based on existing cDNA sequences and, if cDNA sequences were missing or aberrantly spliced, on the known splice pattern of other plant hexokinase genes, provided that the consensus donor and acceptor splice sites are conserved. The predicted transit peptides in the type A hexokinases and the membrane anchors in the type B and D hexokinases are shown as small boxes under exon 1.

We have previously shown that the major hexokinase in *Physcomitrella*, PpHxk1, is responsible for most of the hexokinase activity in protonemal tissue extracts. Thus, 80% of the total glucose phosphorylating activity, including almost all of the activity in the chloroplast stroma, disappears when the *PpHXK1 *gene is disrupted [5]. However, the same experiment also showed that a minor glucose phosphorylating activity which is associated with chloroplast membranes is unaffected by the *PpHXK1 *disruption [5]. We therefore expected that other hexokinases would be responsible for the residual enzymatic activity that is independent of PpHxk1, and in particular for the activity that is associated with the membrane fraction. Consistent with this the genome sequence [31] revealed that there are no less than eleven hexokinase genes in *Physcomitrella *and we found that they can be grouped into four different types that show some variation in their exon-intron organization (Figure 1). This exceeds the number of genes in both *Arabidopsis *(six) and rice (ten). It has previously been noted that metabolic enzymes are overrepresented in *Physcomitrella*, possibly reflecting a more diverse metabolism in mosses than in seed plants [41].

The well-conserved protein sequences and the presence of cDNAs for most of the genes among our PCR products and in public EST data bases [34,42] suggest that they encode functional products which are expressed in protonemal tissue. The only possible exception is *PpHXK6*, for which no transcript has been found. However, for four of the genes, *PpHXK5, PpHXK9, PpHXK10 *and *PpHXK11*, only aberrantly spliced transcripts causing premature termination have been sequenced. It should be noted that two other genes, *PpHXK3 *and *PpHXK7*, had both correctly and incorrectly spliced transcripts. This suggests that alternative splicing is common, and that correctly spliced products therefore could exist also for the four aberrantly spliced genes. A sequence analysis of the genes does not suggest that any of them is a pseudogene, since both predicted protein sequences and other important features such as consensus sites for splicing are well conserved. The only possible exception is PpHxk11 which has a few amino acid substitutions in positions suggested to be important for catalytic activity (see below).

### Two novel types of hexokinases, types C and D, are present in *Physcomitrella*

We have previously classified plant hexokinases into two types [5] depending on their N-terminal sequences which contain either chloroplast transit peptides (type A) or hydrophobic membrane anchors (type B). Furthermore, the membrane anchors of the type B hexokinases are highly conserved between different plant species, suggesting a common evolutionary origin for this sequence [5]. Most of the *Physcomitrella *hexokinases belong to the two previously described types. Thus, in addition to PpHxk1, two more type A hexokinases are encoded by *PpHXK5 *and *PpHXK6*. Based on the sequences, PpHxk6 appears to be more closely related to PpHxk1 than PpHxk5. Four of the predicted *Physcomitrella *hexokinases, PpHxk2, PpHxk3, PpHxk7 and PpHxk8 have N-terminal membrane anchors similar to the N-termini of type B hexokinases from other plants.

However, some of the *Physcomitrella *hexokinases do not conform to the criteria that we used to define types A and B. One hexokinase, PpHxk4, has a truncated N-terminus without either a membrane anchor or an organelle import peptide. We will refer to this novel type as a type C hexokinase. Interestingly, no hexokinase with a truncated N-terminus is encoded by the *Arabidopsis *genome. The rice genome predicts two hexokinases with truncated N-termini, the *OsHXK7 *and *OsHXK8 *gene products [7], but their N-terminal sequences do not resemble PpHxk4. Instead, they look like truncated type B hexokinase membrane anchors, with most of the twelve first amino acid residues being alanines or valines.

**Figure 2:**
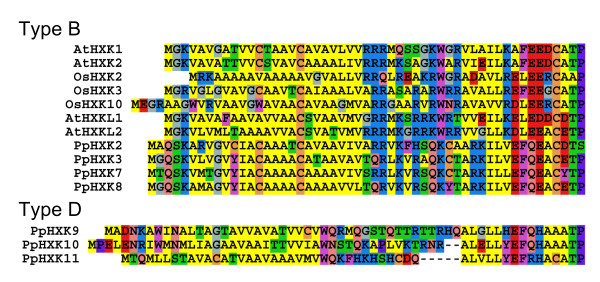
**Comparison of the N-terminal sequences of type B and D hexokinases**. The sequences shown are the N-terminal ends of the proteins. Type B hexokinases from rice, *Arabidopsis *and *Physcomitrella *are shown at the top, and the three *Physcomitrella *type D hexokinases at the bottom. The colour coding used is: L, V, I, M, A - yellow; K, H, R - blue; E, D - red; W, F, Y - magenta; T, S - green; N, Q - pink; G - gray; P - violet; C - orange.

**Figure 3:**
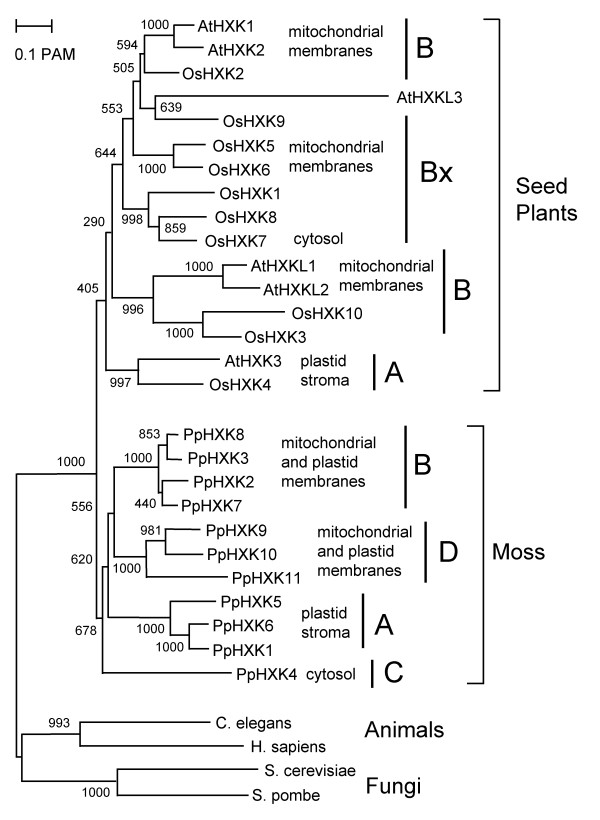
**Phylogenetic tree of plant hexokinases**. The sequences included in the comparison were those predicted by the ten hexokinase-encoding genes in the rice genome, the six hexokinase and hexokinase-like genes in the *Arabidopsis *genome and the eleven *Physcomitrella *hexokinases discussed in the present work. Aligned amino acid sequences corresponding to residues 69-439 in PpHxk1, which excludes the divergent N- and C-termini, were used to calculate a phylogenetic tree as described in Methods. The alignment is shown in additional file 5: Figure S1. Hexokinase sequences from the budding yeast *S. cerevisiae *(ScHxk2), the fission yeast *S. pombe*, the nematode *C. elegans*, and human glucokinase (hexokinase IV) were included to root the tree. The subdivisions of the hexokinases into types A, B, C and D and their intracellular localisation, if known, are also shown. BX stands for seed plant proteins that cluster with the type B hexokinases, but whose N-termini are less conserved. The bar represents a PAM value (percent accepted point mutations) of 10%. The numbers at the branch points are bootstrap values derived from 1000 randomized sequences.

The *Physcomitrella *genome also predicts three additional hexokinases, PpHxk9, PpHxk10, and PpHxk11, which we will refer to as type D. Like the type B hexokinases, they possess N-terminal membrane anchors, but these anchors differ in sequence from the type B hexokinases (Additional file 4: Table S4). Thus, the N-termini of the type B hexokinases from *Arabidopsis*, rice and *Physcomitrella *are more similar to each other than to the N-termini of the type D hexokinases (Figure 2). As discussed below, several other diagnostic motifs, the overall sequence similarity (Figure 3), and the exon-intron structure (Figure 1) also distinguish the type D proteins from the previously described type B hexokinases.

### Conserved motifs and amino acid residues in the *Physcomitrella *hexokinases

The N-termini of the *Physcomitrella *hexokinases were further analyzed using prediction software. As shown in Table 1, TMHMM 2.0 [43] found a single N-terminal transmembrane helix in all four type B hexokinases and all three type D hexokinases, but no helix in any type A or C protein. Consistent with this, TargetP 1.1 [44] predicts a "secretory pathway" location for all type B and D proteins. As previously noted [5], proteins with N-terminal membrane anchors tend to be classified as secretory pathway proteins, since secreted proteins have a hydrophobic signal peptide. As expected, TargetP also predicts that two of the three type A hexokinases (PpHxk1 and PpHxk6) localize to chloroplasts, and the type C hexokinase (PpHxk4) was classified as "other", consistent with a cytosolic location (Table 1). The only unexpected result was that the type A hexokinase PpHxk5 was predicted to localize to mitochondria rather than to chloroplasts, which is inconsistent with our GFP fusion data (see below).

**Table 1:** Predicted intracellular locations and transmembrane helices of moss hexokinases

Protein	Type	**cTP**^**a**^	**mTP**^**a**^	**SP**^**a**^	**other**^**a**^	**Loc**^**a**^	**RC**^**a**^	**TPlen**^**a**^	**TMH**^**b**^	**TMhelix**^**b**^
PpHxk1	A	**0.800**	0.207	0.007	0.046	C	3	37	0	-
PpHxk2	B	0.040	0.129	**0.724**	0.013	S	3	22	1	aa 7-26
PpHxk3	B	0.043	0.091	**0.777**	0.016	S	2	22	1	aa 7-26
PpHxk4	C	0.337	0.196	0.080	**0.452**	-	5	-	0	-
PpHxk5	A	0.120	**0.373**	0.008	0.129	M	4	14	0	-
PpHxk6	A	**0.685**	0.110	0.010	0.043	C	3	44	0	-
PpHxk7	B	0.164	0.043	**0.540**	0.032	S	4	22	1	aa 7-26
PpHxk8	B	0.082	0.062	**0.771**	0.022	S	2	22	1	aa 7-26
PpHxk9	D	0.052	0.067	**0.350**	0.182	S	5	32	1	aa 7-29
PpHxk10	D	0.048	0.031	**0.527**	0.298	S	4	28	1	aa 7-29
PpHxk11	D	0.010	0.064	**0.877**	0.108	S	2	20	1	aa 5-24

^a ^Intracellular locations and target peptides predicted by TargetP 1.1 [44]. Abbreviations: cTP, chloroplast transit peptide; mTP, mitochondrial targeting peptide; SP, secretory pathway signal peptide; other, any other location; Loc, predicted subcellular localization (C, chloroplasts; S, secretory pathway; M, mitochondria); RC, Reliability Class (1 is the most reliable prediction and 5 the weakest); TPlen, predicted target peptide length. For each protein, the predicted location with the highest score is shown in bold style.

^b ^Transmembrane helices predicted by TMHMM 2.0 [43]. Abbreviations: TMH, predicted number of N-terminal transmembrane helices; TMhelix, amino acids predicted to be part of a transmembrane helix.

A number of conserved sequence motifs and structurally or functionally important amino acid residues have been identified by x-ray crystallography and comparisons of hexokinases from different organisms. Bork *et al. *[45,46] described seven conserved regions in hexokinases which they named *phosphate 1*, *sugar binding*, *connect 1*, *phosphate 2*, *helix*, *adenosine *and *connect 2*, based on the known or suspected functions of these regions. Kuser *et al. *([47] Table II) identified 20 amino acid residues that are highly conserved in 317 hexokinases. Mutational and structural studies have shown that the catalytic residue is an aspartic acid (D211 in the yeast hexokinase ScHxk2) whereas four other residues (S158, K176, E269 and E302 in ScHxk2) contribute to hexose binding [48].

First, we note that the catalytic aspartic acid is strictly conserved in all eleven *Physcomitrella *sequences, as are all but one hexose binding residue. The only exception is the K176 in ScHxk2, which is replaced by a glutamic acid in PpHxk11. As for the 20 most conserved residues [48], we note that 19 of them are strongly conserved in all plant hexokinases (the exception is C268 in ScHxk2). Interestingly, these 19 residues are strictly conserved in all *Physcomitrella *sequences except PpHxk11, which has four substitutions (Additional file 5: Figure S1). For comparison, we note that the highly divergent catalytically inactive AtHkl3 protein [13] has 12 substitutions in these 19 positions. This includes the catalytic aspartic acid, which is an asparagine in AtHkl3, and two of the hexose binding residues. The less divergent AtHkl1 and AtHkl2 proteins, also thought to be catalytically inactive, have two and three substitutions, respectively, in the 19 conserved residues, none of which involve the catalytic or hexose binding residues.

An inspection of the seven regions described by Bork *et al. *[46] shows that they all are well conserved in the *Physcomitrella *proteins (Additional file 5: Figure S1). There are however, some noteworthy exceptions. First, the type D hexokinases share several substitutions in the conserved regions which are not found in any other hexokinases. Thus, they have a cysteine followed by a leucine in the *phosphate 1 *motif where most other hexokinases have a valine followed by a glutamine. Furthermore, a phenylalanine in the *sugar binding *motif, which is strictly conserved in all other hexokinases, is replaced by a leucine in the three type D proteins. Finally, the latter also share a deletion of two residues at the end of the *phosphate 2 *motif which is not found in any other hexokinases. None of these changes involve residues shown to be critical for catalytic activity, but it is still possible that they could affect the activity and/or substrate specificity of the type D proteins. In addition to these changes, PpHxk11 has several more substitutions in the conserved regions, consistent with its generally more divergent sequence. Finally, we note that all *Physcomitrella *hexokinases have an insertion in the *adenosine *motif, which is found also in other plant hexokinases [7,13].

### The *Physcomitrella *hexokinases show evidence of concerted evolution

In order to gain a better understanding of how the different hexokinases are related to each other, we used the predicted sequences of the *Arabidopsis*, rice and *Physcomitrella *hexokinases to construct an evolutionary tree. We limited the analysis to these three plant species since their genome sequences have been completed and since the rice and *Arabidopsis *hexokinases already have been fairly well studied [7,13,49]. The variable N-termini and C-termini were excluded from the analysis in order to avoid ambiguities in the sequence alignment, and to ensure that the result would be independent of the N-termini, thus making it possible to assess to what extent the latter have co-evolved with the rest of the proteins (Additional file 5: Figure S1).

The resulting tree is shown in Figure 3. Surprisingly, we found that all eleven *Physcomitrella *hexokinases are more closely related to each other than to other plant hexokinases, thus forming a single branch within the tree. This was unexpected since the *Arabidopsis *and rice sequences do not cluster in this way, but instead are interspersed (Figure 3). This is particularly evident in the case of the type A hexokinases, where the single proteins present in *Arabidopsis *(AtHxk3) and rice (OsHxk4) are more similar to each other than to the other *Arabidopsis *and rice hexokinases (Figure 3). In contrast, the three type A hexokinases in *Physcomitrella*, PpHxk1, PpHxk5 and PpHxk6, are more similar to the other *Physcomitrella *hexokinases than to their orthologs AtHxk3 and OsHxk4. We conclude from this that the *Physcomitrella *hexokinases show evidence of concerted evolution, unlike the *Arabidopsis *and rice proteins.

It should further be noted that within the *Physcomitrella *sequences, the four above described hexokinase types form well-defined branches suggesting a distinct origin for each type. Thus, the three type D hexokinases are clearly more closely related to each other than to the four type B hexokinases, and *vice versa*. This suggests that each type of hexokinases arose from a single ancestral gene, which subsequently underwent duplications. This interpretation is further confirmed by the fact that the moss type B hexokinases have lost intron 2, which is present in the other moss hexokinases, including the type D hexokinases (Figure 1). Finally, we note that the sequence of the type C hexokinase, PpHxk4, is more distantly related to the other *Physcomitrella *hexokinases than they are to each other. This suggests that the type C hexokinase may represent an early branch on the tree, which has been lost in seed plants.

### Intracellular localization of the *Physcomitrella *hexokinases

**Figure 4:**
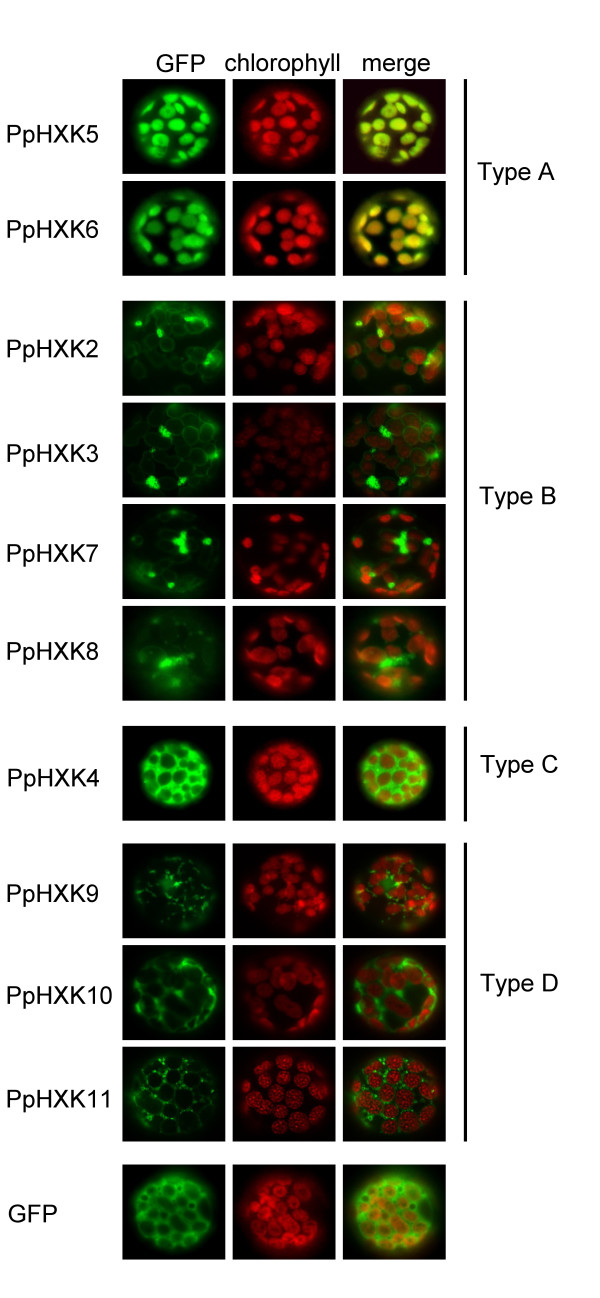
**Intracellular localization of *Physcomitrella *hexokinase-GFP fusions**. Fluorescence microscopy pictures of wild type protoplasts transiently expressing different GFP fusions. GFP fluorescence is shown in green, with the chlorophyll auto-fluorescence in red as a chloroplast marker. Protoplasts expressing GFP alone were also included as a control. The white bars represent 5 μm.

**Figure 5:**
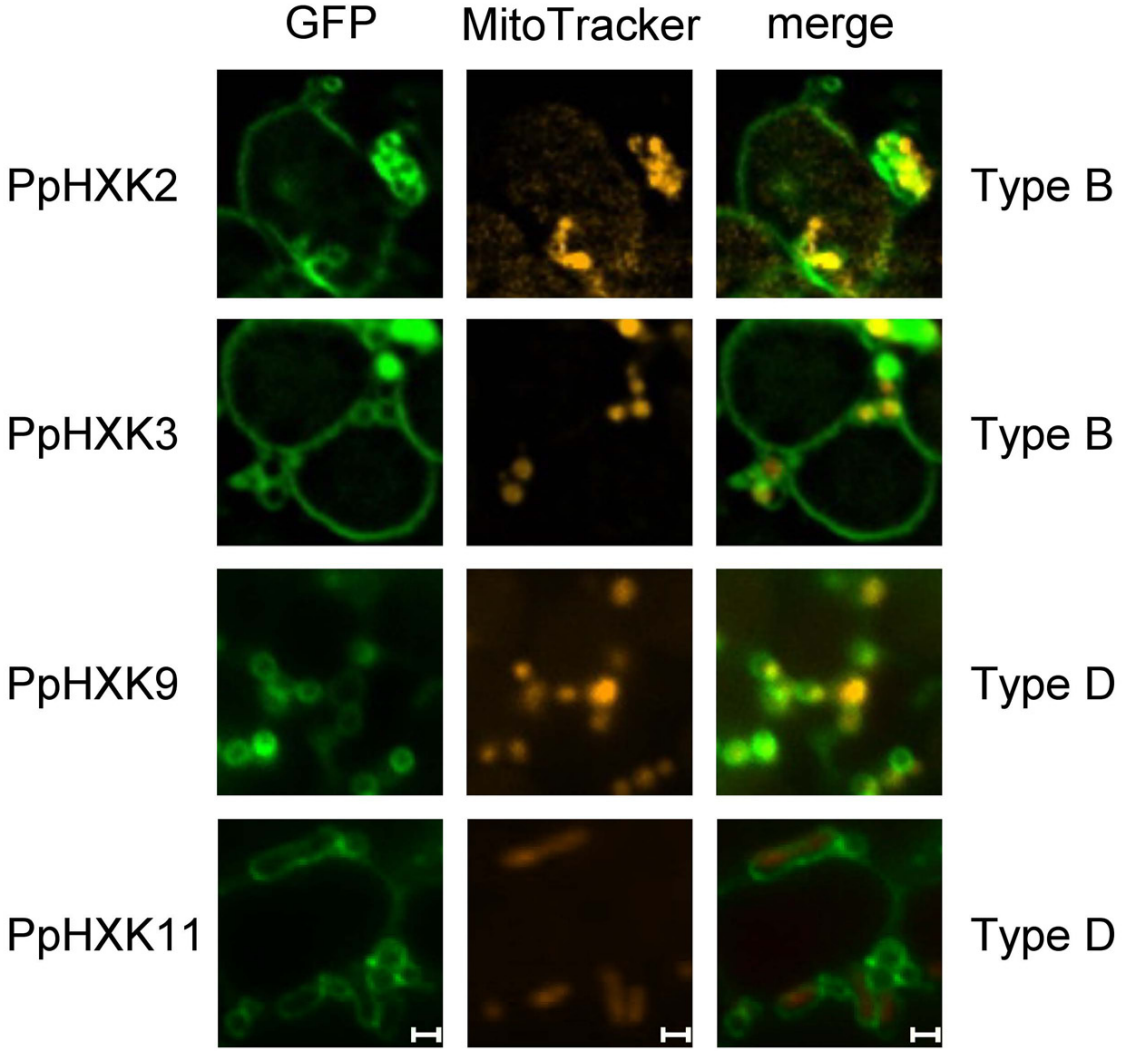
**Localization of *Physcomitrella *hexokinases to mitochondria**. GFP fluorescence is show in green and the mitochondria specific dye MitoTracker^® ^in orange. The white bars represent 1 μm.

We proceeded to study the intracellular locations of the moss hexokinases. Sequences from the new hexokinases, expressed from the 35S promoter, were fused in frame to GFP. These constructs were transiently expressed in *Physcomitrella *protoplasts and the GFP fluorescence was monitored (Figure 4). Based on the sequence similarity of the N-terminal membrane anchors in PpHxk2, PpHxk3, PpHxk7 and PpHxk8 to those found in AtHxk2 (Figure 2) we expected that they would localize to the outer mitochondrial membrane, as shown for AtHxk2 and several other type B hexokinases [7-9,12,13,49]. Consistent with this, we found that the *Physcomitrella *type B hexokinases tested also localize to small ring-like membrane structures (Figure 4) which were identified as mitochondrial membranes by co-staining with MitoTracker^® ^(Figure 5). In contrast, truncated GFP fusions which lacked the membrane anchors showed a diffuse localization throughout the cell (Additional file 6: Figure S2). We conclude that the N-terminal membrane anchors target the proteins to the mitochondria. We further note that the mitochondria often formed aggregates (Figure 5). This may be an artefact caused by protein overexpression, as shown for other membrane-anchored GFP fusions expressed in plants [50]. A similar aggregation of mitochondria was also seen when several of the *Arabidopsis *hexokinase GFP fusions were overexpressed [13].

**Figure 6:**
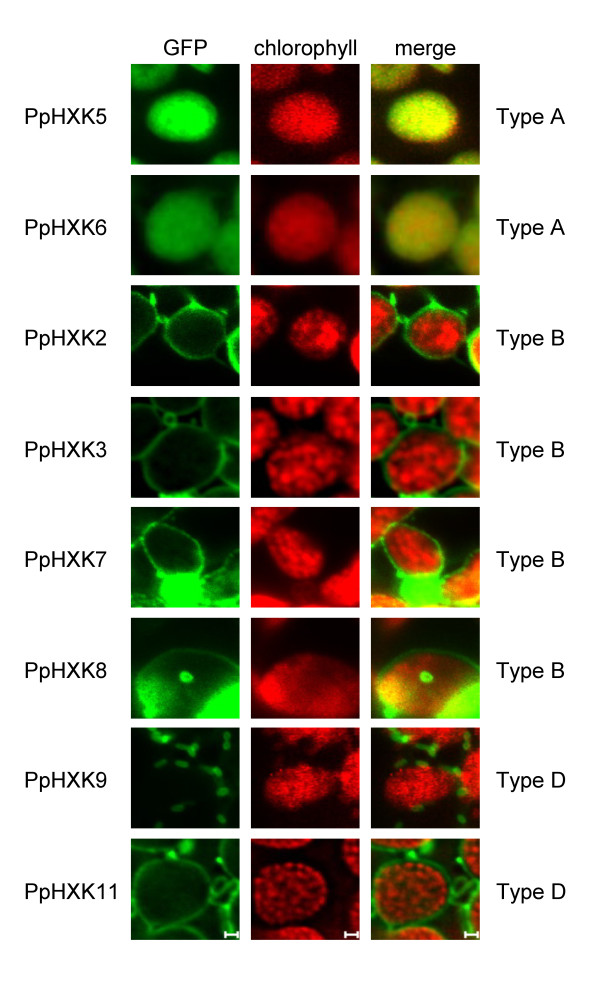
**Localization of *Physcomitrella *hexokinases to chloroplasts**. GFP fluorescence is shown in green and chlorophyll autofluorescence in red. The white bars represent 1 μm.

Surprisingly, the type B hexokinase-GFP fusions also showed fluorescence that was associated with the chloroplast envelope (Figures 4 and 6). This fluorescence was weaker than that being associated with the mitochondria, but it was seen for all four type B hexokinases. This is intriguing since the spinach type B hexokinase SoHxK1 originally was thought to localize to chloroplast envelopes [51]. This finding was, however, challenged by Damari-Weissler *et al. *[9] who reported that SoHxK1 is found only in the outer mitochondrial membrane, with no evidence of a chloroplast localisation. It is conceivable that the hydrophobic anchors in these hexokinases might cause them to adhere non-specifically also to chloroplast membranes. However, we do not think that this is likely since the type D hexokinase PpHxk9 did not show any fluorescence associated with chloroplasts, despite having a membrane anchor and being localized to mitochondria (see below). This suggests that the chloroplast membrane association of some hexokinases is specific. Furthermore, we note that our previous subcellular fractionation revealed that some hexokinase activity is associated with chloroplast membranes, and that this activity, unlike that in the chloroplast stroma, is unaffected by a knockout of *PpHXK1 *[5]. We note that some proteins that are known to target to the chloroplast outer membrane contain N-terminal membrane anchors similar to those found in the type B hexokinases [52].

The three type D hexokinases PpHxk9, PpHxk10 and PpHxk11 also possess membrane anchors and show a similar, though more restricted localisation as the type B proteins. Thus, both PpHxk9 and PpHxk11 localize to the outer mitochondrial membrane, but only PpHxk11 is also associated with the chloroplast envelope, like the type B hexokinases (Figures 4, 5, 6). For PpHxk10, we were unable to clone a *PpHXK10 *full length transcript that was correctly spliced. We therefore made two incomplete PpHxk10-GFP fusions: one containing the entire region encoded by the first exon including the membrane anchor (Figure 4) and one containing the membrane anchor alone. Both fusions localized throughout the cytosol. It is, however, possible that these partial fusions are incorrectly folded due to the hydrophobic nature of the membrane anchor, and that the targeting signal is thus not functional. We cannot therefore rule out that a full-length fusion of PpHxk10 to GFP would localize to the outer mitochondrial membrane, similar to PpHxk9 and PpHxk11.

**Figure 7:**
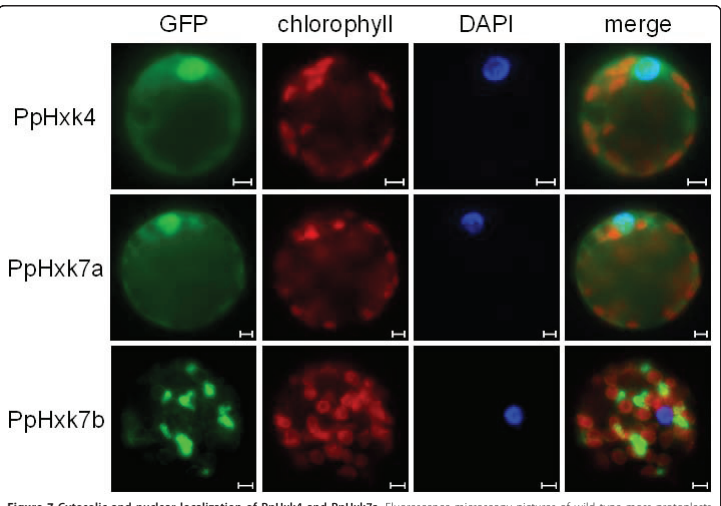
**Cytosolic and nuclear localization of PpHxk4 and PpHxk7a**. Fluorescence microscopy pictures of wild type moss protoplasts transiently expressing PpHxk4, the PpHxk7a splice variant, or the PpHxk7b splice variant fused to GFP. GFP fluorescence is shown in green, with the chlorophyll auto-fluorescence in red as a chloroplast marker. The nucleus is visualized in blue by the fluorescent DNA binding dye DAPI. The white bars represent 5 μm.

In contrast to the above findings, the PpHxk4-GFP fusion shows a diffuse fluorescence throughout the cell, indicating a cytosolic localization (Figure 4) but co-staining with DAPI revealed that it is also enriched in the nucleus (Figure 7). This is similar to what is seen for GFP alone (Figure 4; see also [53]) and is consistent with the absence of either a membrane anchor or a targeting peptide in the N-terminus of PpHxk4. Similar to GFP expressed alone, PpHxk4-GFP is also clearly excluded from the chloroplasts. A likely explanation for this result is that in the absence of specific targeting signals, PpHxk4 is distributed throughout the cytosolic and nuclear compartments. Our finding that *Physcomitrella *possesses a novel type of soluble hexokinase might explain earlier reports of cytosolic hexokinase activities in different plants [54-59]. However, such activities could also be derived from dissociated or alternatively spliced membrane bound hexokinases (see below). That cytosolic hexokinases are likely to exist also in other plants is further suggested by the fact that the glucose which is exported from the chloroplasts after starch degradation would require phosphorylation to be further metabolized [60].

The PpHxk5-GFP and PpHxk6-GFP fusions, finally, had localizations resembling that of PpHxk1 [5]. Thus, we found that they are imported into the chloroplast stroma (Figures 4 and 6). Truncated versions of PpHxk5-GFP and PpHxk6-GFP lacking the transit peptide were evenly distributed in the cytosol, similar to GFP expressed alone (Additional file 6: Figure S2). We conclude that chloroplast import of PpHxk5 and PpHxk6 is dependent of their N-terminal transit peptides, similar to PpHxk1 [5]. Interestingly, a PpHxk5-GFP fusion with a shorter N-terminal truncation of amino acid residues 1-18 is still imported into the chloroplasts, so the targeting information is not immediately adjacent to the N-terminal end of PpHxk5 (Additional file 6: Figure S2).

### PpHxk3 but not PpHxk1 can complement a hexokinase-deficient yeast strain

**Figure 8:**
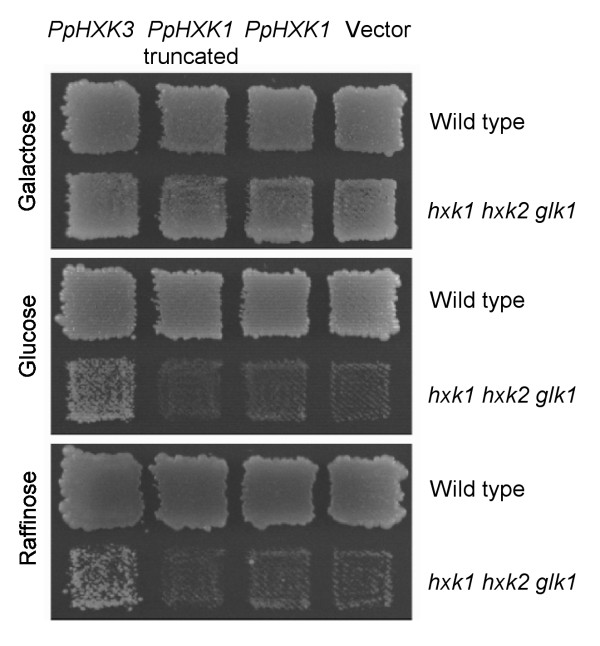
**PpHxk3 can complement a hexokinase-deficient yeast strain**. The picture shows growth of the *hxk1 hxk2 glk1 *triple disrupted yeast strain containing the pFL61 vector with different inserts on plates containing 2% galactose, 2% glucose or 3% raffinose as carbon source. Growth on glucose requires glucokinase activity and growth on raffinose fructokinase activity. The inserts from left to right are: *PpHXK3 *cDNA; *PpHXK1 *cDNA encoding an N-terminally truncated protein; *PpHXK1 *cDNA; no insert.

Several plant hexokinases were cloned by their ability to complement hexokinase-deficient yeast strains [61-63]. We previously found that PpHxk1 fails to complement a *hxk1 hxk2 glk1 *triple mutant yeast strain. We noted that PpHxk1 is a type A hexokinase, while all those that had been shown to work in yeast at that time were type B hexokinases [5]. This prompted us to test if a type B hexokinase from *Physcomitrella *would work in yeast. To this end, we cloned a cDNA encoding PpHxk3 into the yeast shuttle vector pFL61 where the inserts are expressed from the *PGK *promoter. The plasmid was transformed into the *hxk1 hxk2 glk1 *yeast strain and tested for ability to support growth on different carbon sources. As shown in Figure 8, we found that PpHxk3 complements the hexokinase-deficient yeast strain for growth on glucose, which shows that PpHxk3 is expressed and active in yeast. We further found that PpHxk3 can support growth on raffinose, which requires fructokinase activity (Figure 8). This shows that PpHxk3 has a dual specificity for glucose and fructose, similar to PpHxk1 [5]. In contrast, PpHxk1 failed to complement the *hxk1 hxk2 glk1 *triple mutant when expressed from the same vector (Figure 8). To test if this is due to the presence of the chloroplast transit peptide, which might interfere with its function in yeast, we tested a truncated PpHxk1 which lacks residues 1-38. This is the same truncation that causes the PpHxk1-GFP fusion to localize to the cytosol instead of to the chloroplasts [5]. However, the truncated PpHxk1 was still unable to complement the hexokinase-deficient yeast strain (Figure 8). This is in contrast to the type A hexokinases OsHxk4 and LeHxk4 which could complement a hexokinase-deficient yeast strain when their chloroplast transit peptides were deleted [7,12].

### A recent microsatellite mutation in the PpHXK3 gene

During the sequencing of the cDNA and genomic clones we found a polymorphism in an AG microsatellite repeat in the 5'-untranslated region of the *PpHXK3 *gene. The two cDNAs that were sequenced differ by one AG (Additional file 7: Figure S3a), with the shorter variant being present in our genomic clone. We first considered the possibility that two duplicated genes might exist which differ only in this repeat. However, we saw no evidence of this, and only one *PpHXK3 *gene is found in the genome sequence [31]. Interestingly, this gene has the longer variant, unlike our genomic clone. This made us consider the possibility that loss of one AG may have occurred recently in our moss line, which would still be heterogeneous for this mutation, thus explaining the two cDNAs. To test this we cloned two new PCR fragments from the 5'-untranslated region of the *PpHXK3 *gene. Significantly, we found that one has the extra AG and one does not, thus confirming the presence of a polymorphism in our genomic DNA. Two polymorphisms involving microsatellite repeats were also seen in *PpHXK2*, though we did not investigate these as carefully as the mutation in *PpHXK3*. We conclude that sequence evolution by acquisition or loss of microsatellite repeats seems to occur very rapidly in *Physcomitrella*. This could be a consequence of the high frequency of homologous recombination, since unequal sister chromatid exchange and gene conversion, both of which depend on homologous recombination, can generate this kind of polymorphisms.

### Alternative splicing produces a type B hexokinase without a membrane anchor

We found at least one cDNA for ten of the eleven hexokinases in *Physcomitrella*, the only exception being *PpHXK6*. When the cDNA clones were sequenced and compared to other plant hexokinases we found several unexpected splice variants (Additional file 8: Table S5). Thus, we found both intron retention and exon skipping but the most frequent mode of alternative splicing was the use of alternative donor and/or acceptor sites. Most of these aberrantly spliced cDNA sequences would not encode functional hexokinases due to premature termination. The most interesting exception is the *PpHXK7 *cDNA clone pdp03464 that was obtained from the RIKEN bioresource center [34]. PpHxk7 is a type B hexokinase with an N-terminal membrane anchor, but the anchor is not encoded by the alternatively spliced pdp03464 clone (Additional file 7: Figure S3b). In the resulting transcript, the predicted protein instead starts with the methionine codon at position 64. This truncated protein is likely to be functional since the deletion does not affect the phosphate, sugar or adenosine binding domains. Interestingly, we also cloned a normally spliced cDNA from *PpHXK7 *(Additional file 9: Table S6) which encodes a protein with an N-terminal membrane anchor (Additional file 7: Figure S3b). It thus appears that alternative splicing produces two PpHxk7 proteins, one with a membrane anchor and one without it.

Significantly, we found that the splice variant without a membrane anchor, PpHxk7a, localizes to the cytosol and in particular to the nucleus (Figure 7), whereas PpHxk7b localizes to mitochondrial membranes (Figure 7), consistent with the presence of a membrane anchor in that splice variant. It is therefore possible that the PpHxk7a splice variant could be involved in gene regulation. In this context, it should be noted that an artificial deletion of the membrane anchor in the two rice type B hexokinases OsHxk5 and OsHxk6 changed their localization to the nucleus, due to the presence of a cryptic nuclear localization sequence in these proteins [27]. No obvious nuclear localization signal was found in PpHxk7a, but its nuclear localization could be the result of passive diffusion, as is seen also for GFP alone [53]. Our finding suggests the interesting possibility that similar splice variants may exist for type B hexokinases in other plants, and that alternative splicing could provide a general mechanism by which type B hexokinases may enter the nucleus and affect gene expression.

## Discussion

We have previously reported that the major hexokinase in *Physcomitrella*, PpHxk1, which accounts for 80% of the glucose phosphorylating activity, is a novel type of plant hexokinase that is targeted to the chloroplast stroma [5]. We have now extended our study of the hexokinase gene family in *Physcomitrella *by the cloning and characterization of ten new putative hexokinase-encoding genes (Figure 1). An inspection of the encoded protein sequences, in particular the N-termini (Figure 2), suggests that they represent several different types of hexokinases which are targeted to different intracellular compartments.

Four of the moss hexokinases, PpHxk2, PpHxk3, PpHxk7 and PpHxk8, are clearly type B hexokinases since they have N-terminal membrane anchors that are similar in sequence to those found in other type B hexokinases [5]. We therefore expected that GFP fusions to these proteins would localize to mitochondria, as do several type B hexokinases in seed plants [7,8,10,13]. We found that they indeed localize to the outer mitochondrial membrane, but surprisingly, also to the chloroplast envelope (Figs. 5 and 6). A similar dual localization was also seen for one type D hexokinase (PpHxk11). We note that subcellular fractionation data suggested that the spinach type B hexokinase SoHxK1 localizes to the chloroplast envelope [51], but more recent results with GFP fusions suggested that this is not the case [9]. Dual targeting of proteins to mitochondria and chloroplasts has been described in *Physcomitrella *and several other plants, but only for proteins that are targeted to the interior of the organelles [64-66].

PpHxk5 and PpHxk6 are type A hexokinases as evidenced both from the sequence of their N-termini, which resemble organelle import peptides and from the fact that they are closely related to PpHxk1 (Figure 3). Consistent with this, we found that both the PpHxk5-GFP and PpHxk6-GFP fusions localize to the chloroplast stroma (Figure 6). This was surprising in view of our previous finding that a knockout of the *PpHXK1 *gene eliminates all glucose phosphorylating activity in the chloroplast stromal fraction [5]. One possible explanation could be that PpHxk5 and PpHxk6 are minor hexokinases, which contribute only a small part of the total activity, or that they preferentially phosphorylate fructose, an activity which was not abolished in the absence of *PpHXK1 *[5]. An alternative explanation could be that neither PpHxk5 nor PpHxk6 is expressed in the young protonemal tissue used for the subcellular fractionation experiments [5]. Yet another possibility could be that PpHxk5 and PpHxk6 lack hexokinase activity and instead have some other function, as has been suggested to be the case for the Hkl1-3 proteins in *Arabidopsis *[13,67]. Finally, it is possible that *PpHXK5 *and *PpHXK6 *are pseudogenes, since we have not been able to clone any cDNA from *PpHXK6 *and the two cDNAs from PpHXK5 that we sequenced were not correctly spliced. However, we think this is unlikely, since *PpHXK5 *and *PpHXK6 *show no other signs of being pseudogenes.

PpHxk4 represents a novel type of hexokinase, distinct from both types A and B, which we call type C. We base this distinction on two facts. First, the sequence of PpHxk4 shows that it is more distantly related to the type A and B hexokinases in *Physcomitrella *than the latter are to each other. PpHxk4 is thus clearly a separate type of hexokinase. In addition, the truncated N-terminus of PpHxk4 differs both from the membrane anchors found in type B hexokinases and from the organelle targeting peptides found in type A hexokinases. The predicted location of PpHxk4 is in the cytosol, since this is where a protein will end up in the absence of a targeting peptide. Consistent with this, we found that a PpHxk4-GFP fusion shows a diffuse fluorescence throughout the cell, indicating a primarily cytosolic, but also nuclear localization (Figure 7). We further note that PpHxk4 occupies a basal position among the *Physcomitrella *hexokinases, being the most divergent member of the protein family (Figure 3). This suggests that it may represent an ancient type of hexokinase that has been lost in seed plants. Interestingly, while no orthologue of PpHxk4 appears to be present in seed plants, the two rice hexokinases OsHxk7 and OsHxk8 also have truncated N-termini, and OsHxk7 was shown to have a cytosolic localisation [7]. However, the N-termini of OsHxk7 and OsHxk8 look more like truncated versions of the type B hexokinase membrane anchor, with most of the twelve first amino acid residues being hydrophobic. Furthermore, OsHxk7 and OsHxk8 also group together with the type B hexokinases in the phylogenetic tree (Figure 3). Thus, they seem to be divergent type B hexokinases rather than orthologs of the *Physcomitrella *type C hexokinase. Still, it is conceivable that OsHxk7 and OsHxk8 may have a function in rice which is analogous to that of PpHxk4 in moss.

Our finding of a new type of cytosolic hexokinase in *Physcomitrella *is interesting in view of the proposed role of plant hexokinases in glucose sensing and signaling [21,61,68-70]. These discussions have so far focused on membrane integrated type B hexokinases such as AtHxk1 and AtHxk2, which was the only type of plant hexokinase that had been studied prior to the discovery of the type A hexokinases [5]. It has recently been shown that some type B hexokinases can translocate into the nucleus and affect gene expression [25,27]. However, it is not clear how these membrane anchored hexokinases are released from their membrane association and translocated into the nucleus. In contrast, the type C hexokinase PpHxk4, which lacks membrane anchor and is a soluble protein, could more easily move into the nucleus.

In this context, we note that we found evidence that moss type B hexokinases also may translocate to the nucleus. Thus, *PpHXK7 *encodes two differently spliced cDNAs, one of which is missing the membrane anchor (Additional file 7: Figure S3b). The intracellular localization of these two proteins is also very different as seen from the expression of the translational fusions with GFP. In protoplasts expressing the PpHxk7a splice variant that lacks the membrane anchor, the fluorescence is thus localized throughout the cytosol but is also associated with the nucleus (Figure 7). This suggests that alternative splicing could be a molecular mechanism whereby membrane bound type B hexokinases, perhaps also in other plants, may become soluble and thus exert a function inside the nucleus.

The other new type of plant hexokinase, type D, appears to have a similar localization as the type B hexokinases, *i.e. *in the outer mitochondrial membrane and also to some extent in the chloroplast envelope. However, they differ from the type B hexokinases in the sequences of their membrane anchors (Figure 2), and form a distinct clade in the evolutionary tree (Figure 3). Furthermore, they do not share the fusion of exons 2 and 3 that is found in all moss type B hexokinases (Figure 1). Still, the overlapping localizations, and the fact that the type D hexokinases also have membrane anchors, suggests that the type B and D hexokinases may have similar functions. In this context it should be noted that the type B hexokinases is a large and diverse group in seed plants, and that some members have N-termini that are less well conserved (labelled BX in Figure 3). Thus, while no obvious orthologues of the type D hexokinases exist in seed plants, it is conceivable that the more divergent members of the type B group may perform an analogous function as the type D hexokinases do in moss.

It has been proposed that some hexokinases may lack catalytic activity but still have other functions, based on data in fungi [71], flies [72] and plants [13,67]. In particular, three of the six predicted hexokinases in *Arabidopsis*, AtHkl1-AtHkl3, appear to lack glucose phosphorylating activity but are conserved between *A. thaliana *and *A. lyrata*, which suggests that they still are under selection [13]. On the other hand, all ten hexokinases in rice could complement a hexokinase-deficient yeast strain, indicating that they are catalytically active [7]. This raises the question whether non-enzymatic hexokinases are peculiar to *Arabidopsis *or more widespread in plants. The knockout phenotypes and enzymatic activities of PpHxk2-PpHxk11 remain to be determined, but we note that PpHxk2-PpHxk10 are as strongly conserved as PpHxk1, which is an active enzyme [5], suggesting that they also may be active. Consistent with this, we found that PpHxk3 can complement a hexokinase deficient yeast strain (Figure 7). In contrast, PpHxk11 has several substitutions which could affect its activity. With four substitutions in the 19 most conserved residues it is not as divergent as AtHkl3 (12/19), but instead resembles AtHkl1 (3/19) and AtHkl2 (2/19). This is also evident from the tree in Figure 3 where AtHkl3 has a very long branch, whereas AtHkl1, AtHkl2 and PpHxk11 have much shorter branches. It is therefore conceivable that PpHxk11 could have a non-enzymatic function, perhaps in regulation or signaling, as has been suggested for the AtHkl proteins [13].

Surprisingly, we found that the eleven *Physcomitrella *hexokinases are more closely related to each other than to any other plant hexokinase, despite the fact that they represent different types of hexokinases, some of which are found also in seed plants (Figure 3). This is in contrast to the situation in seed plants, where hexokinases of the same type from different plants typically are more closely related to each other than hexokinases of different types from the same plant (ref. [5] and Figure 3). There are at least two possible explanations for this. One is that the different hexokinases in *Physcomitrella *originated by gene duplications after the separation of mosses from seed plants. This is the most straightforward interpretation of the tree in Figure 3, but we do not think that this is a likely explanation since the sequence of the membrane anchor in the type B hexokinases is highly conserved between seed plants and *Physcomitrella *(Figure 2). This suggests a common origin for the latter, since it is unlikely that this unique sequence would have been created twice in evolution just by chance.

A more likely explanation is therefore that several genes encoding different types of hexokinases were present already in the common ancestor of mosses and seed plants, and that these genes co-evolved in *Physcomitrella *by gene conversion [73], making them appear to be more closely related to each other than they really are. It has already been noted that tandemly arrayed genes in *Physcomitrella *are highly similar in sequence, which suggests that they may undergo concerted evolution by gene conversion [74]. The hexokinase genes are not tandemly arrayed, in fact they are all located on different scaffolds in the draft sequence of the *Physcomitrella *genome [31], and those scaffolds that could be linked to the genetic map [75] were all in different linkage groups. However, work in yeast has shown that gene conversion also can be ectopic, *i. e. *take place between related genes on different chromosomes [76]. Such ectopic gene conversion could have provided a mechanism by which the moss hexokinases co-evolved. A testable prediction of this hypothesis is that other dispersed gene families also will show evidence of co-evolution in *Physcomitrella*.

## Conclusions

We have characterized all 11 hexokinase encoding genes in the moss *Physcomitrella *and classified them into different types based on sequence motifs and intracellular localization. We found that the hexokinase gene family is more diverse in *Physcomitrella *than in other plants studied so far, encoding two novel types of hexokinases, types C and D. The presence of a cytoplasmic and nuclear hexokinase (type C) sets *Physcomitrella *apart from vascular plants, and instead resembles yeast, where all hexokinases localize to the cytosol. The fact that all moss hexokinases are more similar to each other than to hexokinases from vascular plants, even though both type A and type B hexokinases are present in all plants, further suggests that the hexokinases in *Physcomitrella *have undergone concerted evolution.

## Authors' contributions

AN, TO, MU and MT carried out the experimental work. TO performed the yeast complementation study. All authors were involved in the sequencing and in the phylogenetic analysis. AN, TO, MU and MT cloned the *Physcomitrella *hexokinases and constructed various plasmids. AN, TO, MU and MT transformed *Physcomitrella *protoplasts and analyzed the GFP expression. AN and MT analyzed the transformed *Physcomitrella *protoplasts treated with the mitochondria specific dye. All authors participated in the design and coordination of the study. All authors have read and approved the final manuscript.

## Supplementary Material

Additional file 1 **Genomic and cDNA clones encoding hexokinases**. *Physcomitrella *hexokinase genes and cDNA clones and the primers used for cloning them into the pCR^®^2.1-TOPO vector. Click here for file

Additional file 2 **Oligonucleotide primers**. Oligonucleotide primers used. Most of the primers are named after the gene to be amplified, whether it binds to the 3' or 5' part, and whether it is followed by a *Bam*HI, *Bgl*II or *Sma*I site. Primers whose names end with a T were used to clone inserts where the membrane anchor or chloroplast transit peptide was removed. The primer combinations used in the various cases are listed in Tables S1 and S3. Click here for file

Additional file 3 **Hexokinase-GFP fusion and yeast expression plasmids**. The first column lists the plasmids used for intracellular localization and yeast complementation studies. The primers and templates used to make these plasmids are listed in the last two columns. The amino acid residues of the different hexokinases that are predicted to be expressed after cloning into the vectors psmRS-GFP (GFP fusions) and pFL61 (yeast complementation) are also listed. Click here for file

Additional file 4 **Sequence Identity matrix for the N-terminal region of type B and D hexokinases**. Comparison of N-terminal regions containing the membrane anchor of type B and D hexokinases, illustrated as a two-way sequence identity matrix. The two or three most similar hexokinases in each comparison are in highlighted in bold. Click here for file

Additional file 5 **Alignment of the hexokinases and hexokinase-like proteins that are predicted by the *Arabidopsis*, rice, and *Physcomitrella *genomes**. The protein sequences shown are those predicted by the annotated genomes. The most common residues in each position are enclosed within boxes. The 20 most conserved residues identified by Kuser *et al. *[47] are marked with asterisks and the seven conserved regions defined by Bork *et al. *[45,46] are also indicated. The core of the alignment, corresponding to amino acid residues 69-439 in PpHxk1, was used to compute the evolutionary tree in Figure 3. Four non-plant hexokinase sequences, from the budding yeast *S. cerevisiae*, the fission yeast *S. pombe*, the nematode *C. elegans *and human hexokinase IV, were included as an outgroup in order to root the tree. Click here for file

Additional file 6 **Intracellular localization of truncated hexokinase-GFP fusions**. Fluorescence microscopy pictures of wild type moss protoplasts transiently expressing different truncated versions of the *Physcomitrella *hexokinases fused to GFP. The hexokinase codons that were fused in frame to GFP are indicated for each hexokinase. GFP fluorescence is shown in green, with chlorophyll auto-fluorescence in red serving as a chloroplast marker. Protoplasts expressing GFP alone were also included as a control. Click here for file

Additional file 7 **Sequence polymorphism in the *PpHXK3 *promoter and alternative splicing of the *PpHXK7 *transcript**. **a**. Microsatellite repeat in the *PpHXK3 *promoter that shows evidence of rapid evolution. The two sequence variants are shown. **b**. The two splice variants of *PpHXK7 *with (*PpHXK7b*) and without (*PpHXK7a*) an N-terminal membrane anchor. The nucleotide sequences and predicted encoded peptide sequences of the two splice variants are shown. The splice sites and the start codons are underlined, and the methionines are highlighted by a black background. Click here for file

Additional file 8 **Alternative splicing of *Physcomitrella *hexokinase transcripts**. The different *Physcomitrella *hexokinase transcripts showing alternative splicing. The plasmids names and the type of alternative splicing within these transcripts are listed together with the predicted effect on the expressed protein. Click here for file

Additional file 9 **Accession numbers for *Physcomitrella *hexokinases**. The accession numbers of the PpHXK2-pPHXK11 transcripts and the corresponding GeneIDs are listed. Click here for file
