# Supplementary material for: Two novel types of hexokinases in the moss Physcomitrella patens
Source: BMC Plant Biol. 2011 Feb 14;11:32. doi: 10.1186/1471-2229-11-32 (PMC3045890; doi:10.1186/1471-2229-11-32)
Supplement: Additional file 1 — Genomic and cDNA clones encoding hexokinases. Physcomitrella hexokinase genes and cDNA clones and the primers used for cloning them into the pCR®2.1-TOPO vector. [file 1471-2229-11-32-S1.PDF]

TABLE S1

Genomic and cDNA clones encoding hexokinases

Plasmid	Insert	bp	PCR primers (see Table S2)[a]
pTO8	PpHXK2 cDNA	2185	PpHXK2-5'B + PpHXK2-3'B
pTO9	PpHXK2 gene	4132	PpHXK2-5'C + PpHXK2-3'C
pTO10	PpHXK3 cDNA	2491	PpHXK3-5'B + PpHXK3-3'B
pTO30	PpHXK3 cDNA	2362	PpHXK3-5'B + PpHXK3-3'B
pTO11	PpHXK3 gene	5210	PpHXK3-5'B + PpHXK3-3'B
pTO12	PpHXK4 cDNA	1972	PpHXK4-5'A + PpHXK4-3'A
pTO13	PpHXK4 gene	3935	PpHXK4-5'A + PpHXK4-3'A
pTO14	PpHXK5 gene	3949	PpHXK5-5'A + PpHXK5-3'A
pdp18063[b]	PpHXK5 cDNA	2446	Not applicable
pdp33748[b]	PpHXK5 cDNA	2876	Not applicable
pMU8	PpHXK6 gene	3091	PpHXK6-F + PpHXK6-R
pdp03464[b]	PpHXK7 cDNA	2318	Not applicable
pAN34	PpHXK7 cDNA	1877	PpHXK7-F + PpHXK7-R
pAN29	PpHXK8 cDNA	1828	PpHXK8-5'A + PpHXK8-3'A
pAN24	PpHXK9 cDNA	2262	PpHXK9-5'A + PpHXK9-3'A
pAN30	PpHXK10 cDNA	2166	PpHXK10-5'B + PpHXK10-3'B
pAN31	PpHXK10 cDNA	1946	PpHXK10-5'B + PpHXK10-3'B
pAN32	PpHXK10 cDNA	1819	PpHXK10-5'B + PpHXK10-3'B
pAN23	PpHXK11 cDNA	1851	PpHXK11-5'A + PpHXK10-3'A

[a] All PCR products were cloned into pCR®2.1-TOPO (Invitrogen).

[b] EST clones from the RIKEN collection [34].
