# Supplementary material for: Two novel types of hexokinases in the moss Physcomitrella patens
Source: BMC Plant Biol. 2011 Feb 14;11:32. doi: 10.1186/1471-2229-11-32 (PMC3045890; doi:10.1186/1471-2229-11-32)
Supplement: Additional file 2 — Oligonucleotide primers. Oligonucleotide primers used. Most of the primers are named after the gene to be amplified, whether it binds to the 3' or 5' part, and whether it is followed by a BamHI, BglII or SmaI site. Primers whose names end with a T were used to clone inserts where the membrane anchor or chloroplast transit peptide was removed. The primer combinations used in the various cases are listed in Tables S1 and S3. [file 1471-2229-11-32-S2.PDF]

TABLE S2

Oligonucleotide primers

Name	Sequence	Used for
PpHXK3-5'B	GAGTTGTGTTGTCGCCGCTGAGTCT	Cloning of PpHxk3
PpHXK3-3'B	GCCAAAAGTCCGGAAAAGCGAATC	Cloning of PpHxk3
PpHXK2-5'B	ACGCGGGGGTCATGGGCTCT	Cloning of PpHxk2
PpHXK2-3'B	TGCCGAGATGCGTGATTA	Cloning of PpHxk2
PpHXK2-5'C	AGATGGCGCAATCGAAAGCC	Cloning of PpHxk2
PpHXK2-3'C	CCTGCCAGTCTAGCACCTCT	Cloning of PpHxk2
PpHXK4-5'A	GGGAGGTTAGTAGCTGCCCAGGGTTA	Cloning of PpHxk4
PpHXK4-3'A	AGGCCATCAGCTGTGACTGCACTTC	Cloning of PpHxk4
PpHXK5-5'A	GGTAAGAAGCAACCGGAGCGATGTC	Cloning of PpHxk5
PpHXK5-3'A	GAGTTGACGATGCCTTGTTCTACGG	Cloning of PpHxk5
PpHXK6-F	TGTAGCTCTAGATTCCTCGGATCTG	Cloning of PpHxk6
PpHXK6-R	CGACCCTAGTCATAAAGATCATATCTGC	Cloning of PpHxk6
PpHXK7-F	CGAGACTTTTTAGAAGAGCTG	Cloning of PpHxk7
PpHXK7-R	TCTCGAAACTATGAGGATAA	Cloning of PpHxk7
PpHXK8-5'A	ACTGGTTCGTGTTCCAGTGTTGAC	Cloning of PpHxk8
PpHXK8-3'A	GCATAAGTAGAGTACAGTCCTAACG	Cloning of PpHxk8
PpHXK9-5'A	GCTCTTGATAATGGCTGGTGGAC	Cloning of PpHxk9
PpHXK9-3'A	TTCAAGGATGGATAACGCACTCTC	Cloning of PpHxk9
PpHXK10-5'B	CAATGCCGGAATTGGAGAATAGG	Cloning of PpHxk10
PpHXK10-3'B	CTTACCTATGCTGGGAGTGACGT	Cloning of PpHxk10
PpHXK11-5'A	AAGTTGTAAAGTAATCTCTCCGTGC	Cloning of PpHxk11
PpHXK11-3'A	ATGTTGTACTGGACCGATGATAGC	Cloning of PpHxk11
PpHXK3-5'BHI	TTGAGGGATCCAGAGAGAGGCAGAAATGG	Fusion of PpHxk3 to GFP
PpHXK3-5'BHI-T	GCGGGGATCCAGCGAATGAAAGTGCGAG	Fusion of PpHxk3 to GFP
PpHXK3-3'BHI	CAAGAGGATCCAGGAGGGCACATACTCGG	Fusion of PpHxk3 to GFP
PpHXK2-5'BHI	AGAGAGGGATCCGGGGAAAGATGGCGCA	Fusion of PpHxk2 to GFP
PpHXK2-5'BHI-T	TGATTGTGGATCCGCGCATGAAGTTTCA	Fusion of PpHxk2 to GFP
PpHXK2-3'BHI	TGGTTTCATCGGATCCAAGAAGAAAGATG	Fusion of PpHxk2 to GFP
PpHXK4-5'BHI	TGCAGCTTCAGAGGATCCCAACATGGAA	Fusion of PpHxk4 to GFP
PpHXK4-3'BHI	ACAATGTCCTTGGATCCACTTGGTGACTCC	Fusion of PpHxk4 to GFP
PpHXK5-5'BHI	TAAGAAGGATCCGGAGCGATGTCGATGA	Fusion of PpHxk5 to GFP
PpHXK5-5'BHI-T	CGAGGGTGGGATCCGTGATGTCTCCATG	Fusion of PpHxk5 to GFP
PpHXK5-3'BHI	CTCTAAGTGGATCCCCATCTCCCCTGATT	Fusion of PpHxk5 to GFP
PpHXK6-5'BHI	GCTCTGGATCCCTCGGATCTGG	Fusion of PpHxk6 to GFP
PpHXK6-5'BHI-T	CGTTTCATGGATCCGGGATGG	Fusion of PpHxk6 to GFP
PpHXK6-3'BHI	GGATCCCCCCAGAAGGCAACTCC	Fusion of PpHxk6 to GFP
pdp03464-BglII-F	AGATCTTATGGCGGTCGAGATGCACG	Fusion of PpHxk7 to GFP
pdp03464-BglII-R	AGATCTTGACGTACTCTGCATGTGATGCA	Fusion of PpHxk7 to GFP
PpHXK7-BglII	AAGATCTAATGACACAATCGAAGGTAATG	Fusion of PpHxk7 to GFP
PpHXK8-5'BHI	GGATCCATGGGGCAATCGAAAGC	Fusion of PpHxk8 to GFP
PpHXK8-5'BHI-T	GGATCCATGCGAGTGAAAGTCCGATCG	Fusion of PpHxk8 to GFP
PpHXK8-3'BHI	AAGGATCCCGTCTGGCAAGTGGTC	Fusion of PpHxk8 to GFP
PpHXK9-5'BHI	CAAGCTTGTGCAGGATCCCTTGTCGAG	Fusion of PpHxk9 to GFP
PpHXK9-5'BHI-T	GGCAGAGGATCCAGATGAGCACACAAAC	Fusion of PpHxk9 to GFP

PpHXK9-3'BHI	ATGTGGGCGATCGGATCCCGTCCGG	Fusion of PpHxk9 to GFP
PpHXK10-5'BHI	GGATCCATGCCGGAATTGGAGAATAGG	Fusion of PpHxk10 to GFP
PpHXK10-5'BHI-T	GGATCCATGAGTACACAGAAGGCCCCTCTC	Fusion of PpHxk10 to GFP
PpHXK10-3'BHI	GGATCCCGTCCGGAAGATTCTCCACGTAAG	Fusion of PpHxk10 to GFP
PpHXK10-3'BHI-2	GGATCCTCCAGGCAATCACCACGGTA	Fusion of PpHxk10 to GFP
PpHXK11-5'BHI	TTTCGATGATTCGGATCCCTTGTAGGAGC	Fusion of PpHxk11 to GFP
PpHXK11-5'BHI-T	AGCCGTGGCGGATCCCGTCATGGTCT	Fusion of PpHxk11 to GFP
PpHXK11-3'BHI	AGAAAGGAATGGATCCCCATCTTGCCGCT	Fusion of PpHxk11 to GFP
PpHXK3A-5'SmaI	TTGAGAGCCCGGGAGAGAGGCAGAAATGG	Yeast complementation
PpHXK3A-3'SmaI	ATTTGACCCCCGGGAATTTTAATCAATA	Yeast complementation
PpHXK1-5'SmaI	AACCAGCGCCCGGGTAGCAATGGCGATC	Yeast complementation
PpHXK1-5'SmaI-T	GGAAAGCCCGGGTCTATGTCGGTTCAGA	Yeast complementation
PpHXK1-3'SmaI	AGCTTTGTGCCCGGGTTGTCCTATTTGAAG	Yeast complementation
