# Supplementary material for: Two novel types of hexokinases in the moss Physcomitrella patens
Source: BMC Plant Biol. 2011 Feb 14;11:32. doi: 10.1186/1471-2229-11-32 (PMC3045890; doi:10.1186/1471-2229-11-32)
Supplement: Additional file 3 — Hexokinase-GFP fusion and yeast expression plasmids. The first column lists the plasmids used for intracellular localization and yeast complementation studies. The primers and templates used to make these plasmids are listed in the last two columns. The amino acid residues of the different hexokinases that are predicted to be expressed after cloning into the vectors psmRS-GFP (GFP fusions) and pFL61 (yeast complementation) are also listed. [file 1471-2229-11-32-S3.PDF]

TABLE S3

Hexokinase-GFP fusions and yeast expression plasmids

Plasmid	Insert (codons)	PCR primers (see Table S2)	Template
pMT171	*PpHXK2* (1-521)	PpHXK2-5'BHI + PpHXK2-3'BHI	pTO8
pMT172	*PpHXK2* (30-521)	PpHXK2-5'BHI-T + PpHXK2-3'BHI	pTO8
pTO26	*PpHXK3* (1-517)	PpHXK3-5'BHI + PpHXK3-3'BHI	pTO10
pTO27	*PpHXK3* (30-517)	PpHXK3-5'BHI-T + PpHXK3-3'BHI	pTO10
pTO29	*PpHXK4* (1-489)	PpHXK4-5'BHI + PpHXK4-3'BHI	pTO12
pAN16	*PpHXK5* (1-299)	PpHXK5-5'BHI + PpHXK5-3'BHI	pdp18063
pAN18	*PpHXK5* (31-299)	PpHXK5-5'BHI-T + PpHXK5-3'BHI	pdp18063
pMU1	*PpHXK6* (1-113)	PpHXK6-5'BHI + PpHXK6-3'BHI	pMU8
pMU2	*PpHXK6* (28-113)	PpHXK6-5'BHI-T + PpHXK6-3'BHI	pMU8
pAN35	*PpHXK7* (64-521)	pdp03464-BglII-F + pdp03464-BglII-R	pdp03464
pAN39	*PpHXK7* (1-521)	PpHXK7-BglII + pdp03464-BglII-R	pAN34
pMU3	*PpHXK8* (1-93)	PpHXK8-5'BHI + PpHXK8-3'BHI	pAN29
pMU4	*PpHXK8* (30-93)	PpHXK8-5'BHI-T + PpHXK8-3'BHI	pAN29
pAN43	*PpHXK9* (1-98)	PpHXK9-5'BHI + PpHXK9-3'BHI	pAN24
pAN44	*PpHXK9* (32-98)	PpHXK9-5'BHI-T + PpHXK9-3'BHI	pAN24
pMU5	*PpHXK10* (1-98)	PpHXK10-5'BHI + PpHXK10-3'BHI	pAN30
pMU6	*PpHXK10* (32-98)	PpHXK10-5'BHI-T + PpHXK10-3'BHI	pAN30
pMU7	*PpHXK10* (1-29)	PpHXK10-5'BHI + PpHXK10-3'BHI-2	pAN30
pAN45	*PpHXK11* (1-280)	PpHXK11-5'BHI + PpHXK11-3'BHI	pAN23
pAN46	*PpHXK11* (23-280)	PpHXK11-5'BHI-T + PpHXK11-3'BHI	pAN23
pTO21	*PpHXK1* (1-513)	PpHXK1-5'SmaI + PpHXK1-3'SmaI	pTO5[a]
pTO23	*PpHXK1* (39-513)	PpHXK1-5'SmaI-T + PpHXK1-3'SmaI	pTO5[a]
pTO19	*PpHXK3* (1-517)	PpHXK3A-5'SmaI + PpHXK3A-3'SmaI	pTO10

All inserts were cloned into psmRS-GFP [35] except for pTO21, pTO23 and pTO19, which were based on the yeast shuttle vector pFL61 [38].
[a] Olsson *et al.* 2003 [5].
