# Supplementary material for: Two novel types of hexokinases in the moss Physcomitrella patens
Source: BMC Plant Biol. 2011 Feb 14;11:32. doi: 10.1186/1471-2229-11-32 (PMC3045890; doi:10.1186/1471-2229-11-32)
Supplement: Additional file 4 — Sequence Identity matrix for the N-terminal region of type B and D hexokinases. Comparison of N-terminal regions containing the membrane anchor of type B and D hexokinases, illustrated as a two-way sequence identity matrix. The two or three most similar hexokinases in each comparison are in highlighted in bold. [file 1471-2229-11-32-S4.PDF]

TABLE S4

Sequence Identity matrix for the N-terminal regions of the *Physcomitrella* type B and type D hexokinases

	PpHXK2	PpHXK3	PpHXK7	PpHXK8	PpHXK9	PpHXK10	PpHXK11
PpHXK2	-	**0.698**	**0.716**	**0.698**	0.172	0.150	0.250
PpHXK3	**0.698**	-	**0.811**	**0.830**	0.189	0.183	0.250
PpHXK7	**0.716**	**0.811**	-	**0.792**	0.189	0.200	0.250
PpHXK8	**0.698**	**0.830**	**0.792**	-	0.206	0.200	0.285
PpHXK9	0.172	0.189	0.189	0.206	-	**0.466**	**0.344**
PpHXK10	0.150	0.183	0.200	0.200	**0.466**	-	**0.344**
PpHXK11	0.250	0.250	0.250	0.285	**0.344**	**0.344**	-
