# Supplementary material for: Two novel types of hexokinases in the moss Physcomitrella patens
Source: BMC Plant Biol. 2011 Feb 14;11:32. doi: 10.1186/1471-2229-11-32 (PMC3045890; doi:10.1186/1471-2229-11-32)
Supplement: Additional file 5 — Alignment of the hexokinases and hexokinase-like proteins that are predicted by the Arabidopsis, rice, and Physcomitrella genomes. The protein sequences shown are those predicted by the annotated genomes. The most common residues in each position are enclosed within boxes. The 20 most conserved residues identified by Kuser et al. [47] are marked with asterisks and the seven conserved regions defined by Bork et al. [45,46] are also indicated. The core of the alignment, corresponding to amino acid residues 69-439 in PpHxk1, was used to compute the evolutionary tree in Figure 3. Four non-plant hexokinase sequences, from the budding yeast S. cerevisiae, the fission yeast S. pombe, the nematode C. elegans and human hexokinase IV, were included as an outgroup in order to root the tree. [file 1471-2229-11-32-S5.PDF]

```
AtHXK1    1 ---------------------MGKVAVGATVVCTAAVCAVAVLVVRRRMQSSGKWGRVLAILKAFEEDCATPISKLRQVADA 61
AtHXK2    1 ---------------------MGKVAVATTVVCSVAVCAAAALIVRRRMKSAGKWARVIEILKAFEEDCATPIAKLRQVADA 61
OsHXK2    1 ------------------------MRKAAAAAVAAAAAVGVALLVRRQLREAKRWGRADAVLRELEERCAAPPGRLRQVADA 58
OsHXK6    1 -------------MGKGTVVGTAVVVCAAAAAAVGVAVVVSRRRRSKREAEEERRRRAAAVIEEVEQRFSTPTALLRGIADA 69
OsHXK9    1 -------------------MRKAAALASAAMAAAAVAVVSTVLHQRQRRAAKRSERAEAVLLRDLQERCAAPVELLRQVADA 63
AtHXKL3   1 --------------------MTRKEVVLAVTAATITAVAAGVLMGRWIRRKERRLKHTQRILRKFARECATPVSKLWAVADA 62
OsHXK5    1 ------------MGKAAAVGTAVVVAAAVGVAVVLARRRRRRDLELVEGAAAERKRKVAAVIEDVEHALSTPTALLRGISDA 70
OsHXK7    1 -------------------------------------------------MVAAAVAAAEQVVAALREECATPAARLDGVAAA 33
OsHXK8    1 ----------------------------------------------------MAAVEAEKVVAELRERCATRASLLRDVAAA 30
OsHXK1    1 ----------------MAAAAVAADQKVVTMTSLREGCACAAPPAAAAPPM-PKMAAAQRVVAELREACATPAARLAEVAAA 65
AtHXK3    1 ------------------MSLMFSSPVVTPALGSFTFSSRPRSNYIVMSAVRSNSASTCPILTKFQKDCATPTPYLRNVANA 64
OsHXK4    1 -----------MSAAAAIASPIPAAIAVVQQQRRGRSRGGGSGAAAVRCSAVAPTSAIAPILADLRLRCAAPLPVLRRVADA 71
PpHXK4    1 ----------------------------------------------MENLKNSESCDPSSFLRHFRKSSATPVMLLRHIAQA 36
PpHXK6    1 MRAMAIGSVLGCVGLQFSAVPTFHGSGVASPKMRTQCRAWRRTMSMSVQQPSKRVQRAETLLHDFRRSSATPLPLLHLVADA 82
PpHXK1    1 -------MAIGKVLGCAGFQHSAVPTLREPVRLRAQCRRRGKTVSMSVQKTSKTVQQAEKMLQEFRQSSSTPLPLLHQVADA 75
PpHXK5    1 --MSMTVERVSLFSRGAGVTMHGLLSARVQCVRSPWWGLPKVAGATPNANRVVRVHNADRLVHGLRLAAATPLPLLRQVADA 80
PpHXK9    1 --------------MADNKAWINALTAGTAVVAVATVVCVWQRMQGSTQTTRTTRHQALGLLHEFQHAAATPLYVLRQISEH 68
PpHXK10   1 --------------MPELENRIWMNMLIAGAAVAAITTVVIAWNSTQKAPLVKTRNRALELLYEFQHAAATPLHALRQIAEH 68
PpHXK11   1 ---------------------MTQMLLSTAVACATVAAVAAAVMVWQKFHKHSHCDQALVLLYEFRHACATPLYVLRHISEH 61
PpHXK2    1 -------------------MAQSKARVGVCIACAAATCAVAAVIVARRVKFHSQKCAARKILVEFQEACDTSLLRLRMVVDA 63
PpHXK3    1 -------------------MGQSKVLVGVYIACAAAACATAAVAVTQRLKVRAQKCTARKILVEFQEACETPLPRLRQVVDA 63
PpHXK7    1 -------------------MTQSKVMTGVYIACAAAACAAAAVIVSRRLKVRSQKCTARKILLEFQEACYTPLARLRQVVDA 63
PpHXK8    1 -------------------MGQSKAMAGVYIACAAAACAAAAVVLTQRVKVRSQKYTARKILVEFQEACETPLPRLRQVVDA 63
AtHXKL1   1 ---------------------MGKVAVAFAAVAVVAACSVAAVMVGRRMKSRRKWRTVVEILKELEDDCDTPVGRLRQVVDA 61
AtHXKL2   1 ---------------------MGKVLVMLTAAAAVVACSVATVMVRRRMKGRRKWRRVVGLLKDLEEACETPLGRLRQMVDA 61
OsHXK10   1 -----------------MEGRAAGWVRVAAVGWAVAACAVAAGMVARRGAARVRWNRAVAVVRDLEERCATPAELLQRVVNS 65
OsHXK3    1 ---------------------MGRVGLGVAVGCAAVTCAIAAALVARRASARARWRRAVALLREFEEGCATPPARLRQVVDA 61
ScHXK2    1 -----------------------------------MVHLGPKKPQARKGSMADVPKELMQQIENFEKIFTVPTETLQAVTKH 47
S pombe   1 -------------------------------MSLHDAYHWPSRTPSRKGSNIKLNKTLQDHLDELEEQFTIPTELLHRVTDR 51
Nematode  1 -----------------------MSSIVCHPILDSQNGIATDAVIKKQRPPPLVLSRPKDLIQEACQRLVLSDQQLRRIMQS 59
Human     1 ----------------------------------------------MLDDRARMEAAKKEKVEQILAEFQLQEEDLKKVMRR 36
```

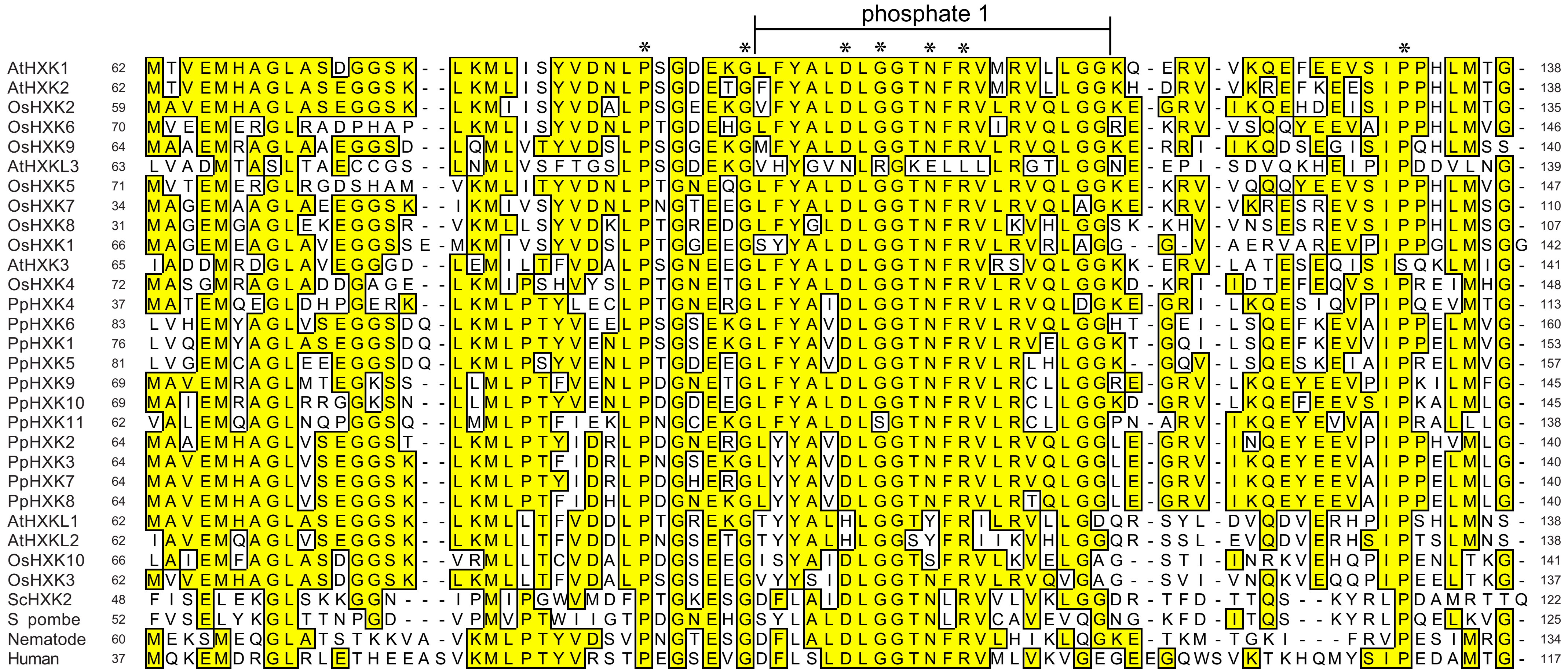
phosphate 1
AtHXK1 62 138
AtHXK2 62 138
OsHXK2 59 135
OsHXK6 70 146
OsHXK9 64 140
AtHXKL3 63 139
OsHXK5 71 147
OsHXK7 34 110
OsHXK8 31 107
OsHXK1 66 142
AtHXK3 65 141
OsHXK4 72 148
PpHXK4 37 113
PpHXK6 83 160
PpHXK1 76 153
PpHXK5 81 157
PpHXK9 69 145
PpHXK10 69 145
PpHXK11 62 138
PpHXK2 64 140
PpHXK3 64 140
PpHXK7 64 140
PpHXK8 64 140
AtHXKL1 62 138
AtHXKL2 62 138
OsHXK10 66 141
OsHXK3 62 137
ScHXK2 48 122
S pombe 52 125
Nematode 60 134
Human 37 117

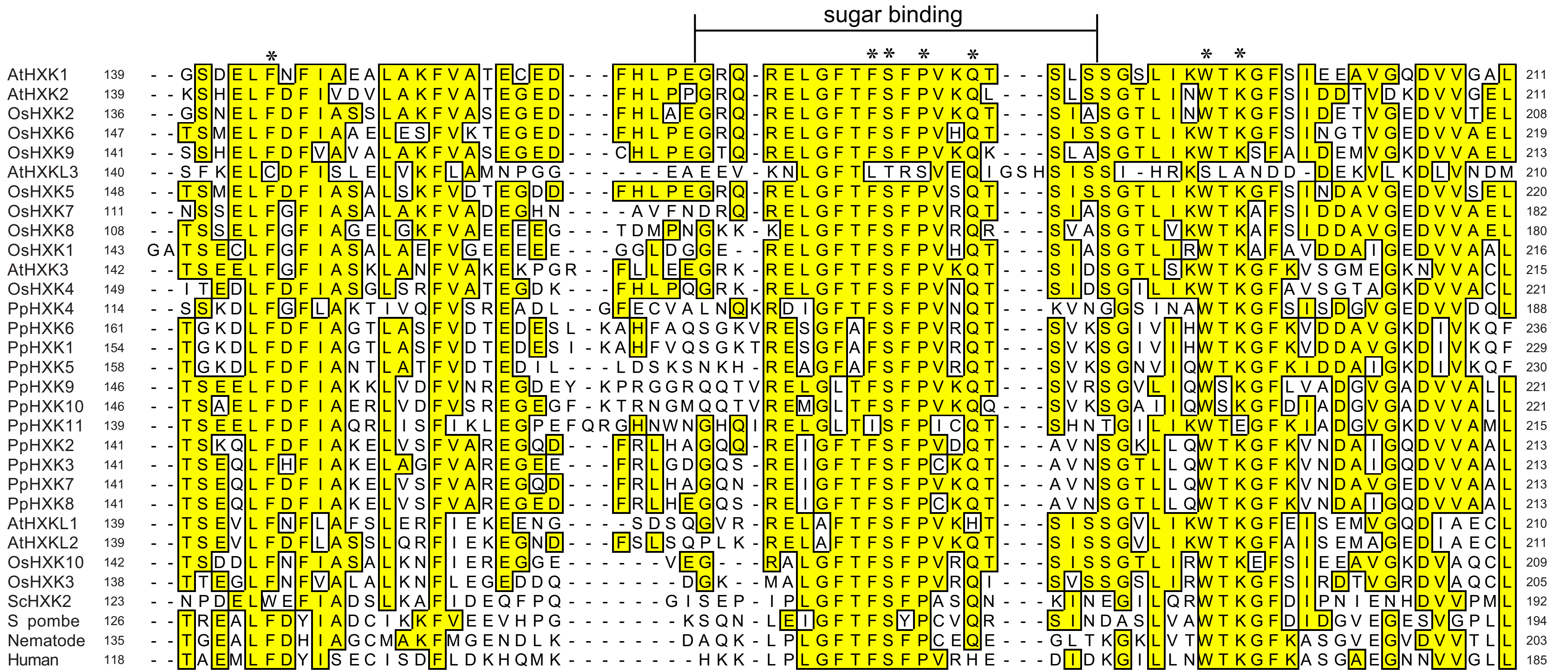
sugar binding
AtHXK1 139 --GSDELFNFIAEALAKFVATECED---FHLPEGRQ-RELGFTFSFPVKQT---SLSSGSLIKWTKGFSIEEAVGQDVVGAL 211
AtHXK2 139 --KSHELFDFIVDVLAKFVATEGED---FHLPPGRQ-RELGFTFSFPVKQL---SLSSGTLINWTKGFSIDDTVDKDVVGEL 211
OsHXK2 136 --GSNELFDFIASSLAKFVASEGED---FHLAEGRQ-RELGFTFSFPVKQT---SIASGTLINWTKGFSIDETVGEDVVTEL 208
OsHXK6 147 --TSMELFDFIAAELESFVKTEGED---FHLPEGRQ-RELGFTFSFPVHQT---SISSGTLIKWTKGFSINGTVGEDVVAEL 219
OsHXK9 141 --SSHELFDFVAVALAKFVASEGED---CHLPEGTQ-RELGFTFSFPVKQK---SLASGTLIKWTKSFAIDEMVGKDVVAEL 213
AtHXKL3 140 --SFKELCDFISLELVKFLAMNPGG------EAEEV-KNLGFTLTRSVEQIGSHSISSI-HRKSLANDD-DEKVLKDLVNDM 210
OsHXK5 148 --TSMELFDFIASALSKFVDTEGDD---FHLPEGRQ-RELGFTFSFPVSQT---SISSGTLIKWTKGFSINDAVGEDVVSEL 220
OsHXK7 111 --NSSELFGFIASALAKFVADEGHN----AVFNDRQ-RELGFTFSFPVRQT---SIASGTLIKWTKAFSIDDAVGEDVVAEL 182
OsHXK8 108 --TSSELFGFIAGELGKFVAEEEEG---TDMPNGKK-KELGFTFSFPVRQR---SVASGTLVKWTKAFSIDDAVGEDVVAEL 180
OsHXK1 143 GATSECLFGFIASALAEFVGEEEEE---GGLDGGE--RELGFTFSFPVHQT---SIASGTLIRWTKAFAVDDAIGEDVVAAL 216
AtHXK3 142 --TSEELFGFIASKLANFVAKEKPGR--FLLEEGRK-RELGFTFSFPVKQT---SIDSGTLSKWTKGFKVSGMEGKNVVACL 215
OsHXK4 149 --ITEDLFDFIASGLSRFVATEGDK---FHLPQGRK-RELGFTFSFPVNQT---SIDSGILIKWTKGFAVSGTAGKDVVACL 221
PpHXK4 114 --SSKDLFGFLAKTIVQFVSREADL--GFECVALNQKRDIGFTFSFPVNQT---KVNGGSINAWTKGFSISDGVGEDVVDQL 188
PpHXK6 161 --TGKDLFDFIAGTLASFVDTEDESL-KAHFAQSGKVRESGFAFSFPVRQT---SVKSGIVIHWTKGFKVDDAVGKDIVKQF 236
PpHXK1 154 --TGKDLFDFIAGTLASFVDTEDESI-KAHFVQSGKTRESGFAFSFPVRQT---SVKSGIVIHWTKGFKVDDAVGKDIVKQF 229
PpHXK5 158 --TGKDLFDFIANTLATFVDTEDIL---LDSKSNKH-REAGFAFSFPVRQT---SVKSGNVIQWTKGFKIDDAIGKDIVKQF 230
PpHXK9 146 --TSEELFDFIAKKLVDFVNREGDEY-KPRGGRQQTVRELGLTFSFPVKQT---SVRSGVLIQWSKGFLVADGVGADVVALL 221
PpHXK10 146 --TSAELFDFIAERLVDFVSREGEGF-KTRNGMQQTVREMGLTFSFPVKQQ---SVKSGAIIQWSKGFDIADGVGADVVALL 221
PpHXK11 139 --TSEELFDFIAQRLISFIKLEGPEFQRGHNWNGHQIRELGLTISFPICQT---SHNTGILIKWTEGFKIADGVGKDVVAML 215
PpHXK2 141 --TSKQLFDFIAKELVSFVAREGQD---FRLHAGQQ-REIGFTFSFPVDQT---AVNSGKLLQWTKGFKVNDAIGQDVVAAL 213
PpHXK3 141 --TSEQLFHFIAKELAGFVAREGEE---FRLGDGQS-REIGFTFSFPCKQT---AVNSGTLLQWTKGFKVNDAIGQDVVAAL 213
PpHXK7 141 --TSEQLFDFIAKELVSFVAREGQD---FRLHAGQN-REIGFTFSFPVKQT---AVNSGTLLQWTKGFKVNDAVGEDVVAAL 213
PpHXK8 141 --TSEQLFDFIAKELVSFVAREGED---FRLHEGQS-REIGFTFSFPCKQT---AVNSGTLLQWTKGFKVNDAIGQDVVAAL 213
AtHXKL1 139 --TSEVLFNFLAFSLERFIEKEENG----SDSQGVR-RELAFTFSFPVKHT---SISSGVLIKWTKGFEISEMVGQDIAECL 210
AtHXKL2 139 --TSEVLFDFLASSLQRFIEKEGND---FSLSQPLK-RELAFTFSFPVKQT---SISSGVLIKWTKGFAISEMAGEDIAECL 211
OsHXK10 142 --TSDDLFNFIASALKNFIEREGGE------VEG---RALGFTFSFPVRQT---SISSGTLIRWTKEFSIEEAVGKDVAQCL 209
OsHXK3 138 --TTEGLFNFVALALKNFLEGEDDQ-------DGK--MALGFTFSFPVRQI---SVSSGSLIRWTKGFSIRDTVGRDVAQCL 205
ScHXK2 123 --NPDELWEFIADSLKAFIDEQFPQ------GISEP-IPLGFTFSFPASQN---KINEGILQRWTKGFDIPNIENHDVVPML 192
S pombe 126 --TREALFDYIADCIKKFVEEVHPG-------KSQN-LEIGFTFSYPCVQR---SINDASLVAWTKGFDIDGVEGESVGPLL 194
Nematode 135 --TGEALFDHIAGCMAKFMGENDLK-------DAQK-LPLGFTFSFPCEQE---GLTKGKLVTWTKGFKASGVEGVDVVTLL 203
Human 118 --TAEMLFDYISECISDFLDKHQMK--------HKK-LPLGFTFSFPVRHE---DIDKGILLNWTKGFKASGAEGNNVVGLL 185

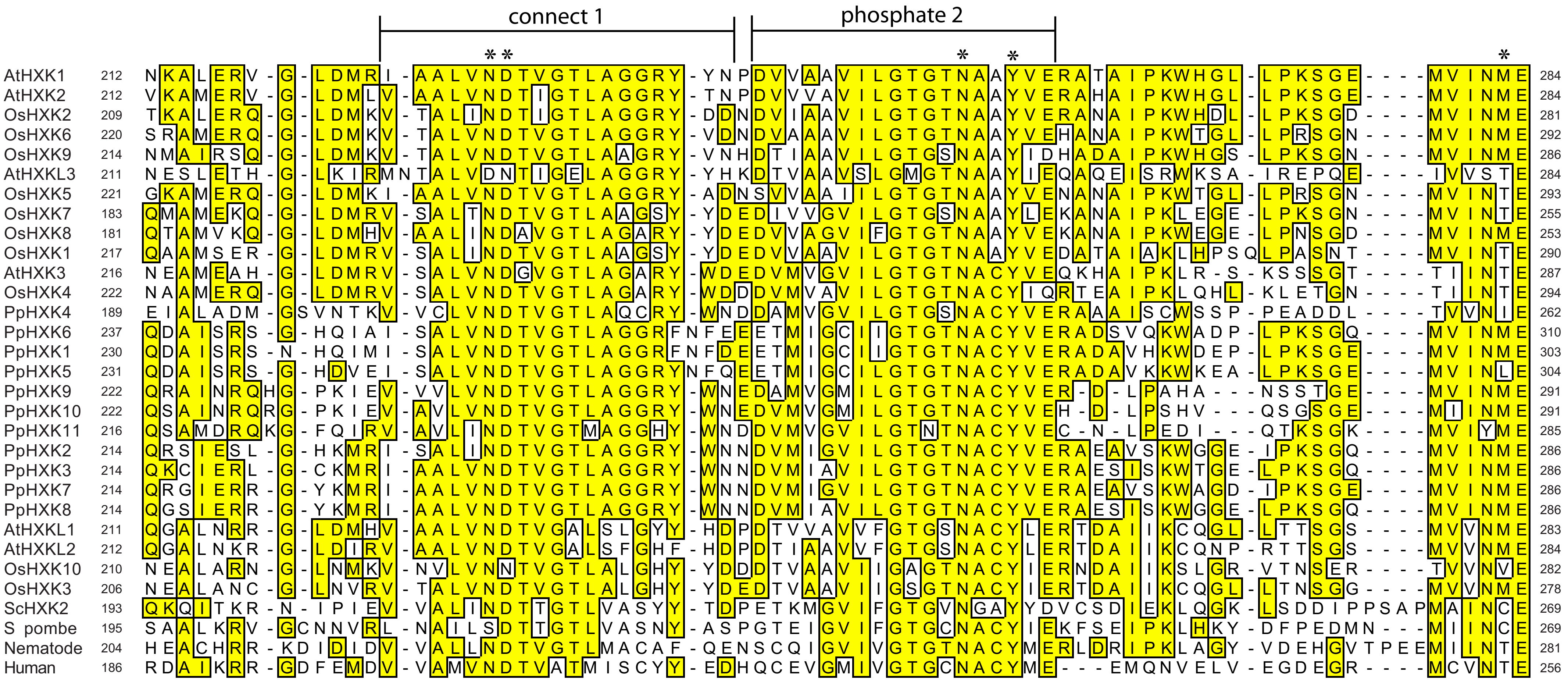
connect 1
phosphate 2
AtHXK1 212 284
AtHXK2 212 284
OsHXK2 209 281
OsHXK6 220 292
OsHXK9 214 286
AtHXKL3 211 284
OsHXK5 221 293
OsHXK7 183 255
OsHXK8 181 253
OsHXK1 217 290
AtHXK3 216 287
OsHXK4 222 294
PpHXK4 189 262
PpHXK6 237 310
PpHXK1 230 303
PpHXK5 231 304
PpHXK9 222 291
PpHXK10 222 291
PpHXK11 216 285
PpHXK2 214 286
PpHXK3 214 286
PpHXK7 214 286
PpHXK8 214 286
AtHXKL1 211 283
AtHXKL2 212 284
OsHXK10 210 282
OsHXK3 206 278
ScHXK2 193 269
S pombe 195 269
Nematode 204 281
Human 186 256

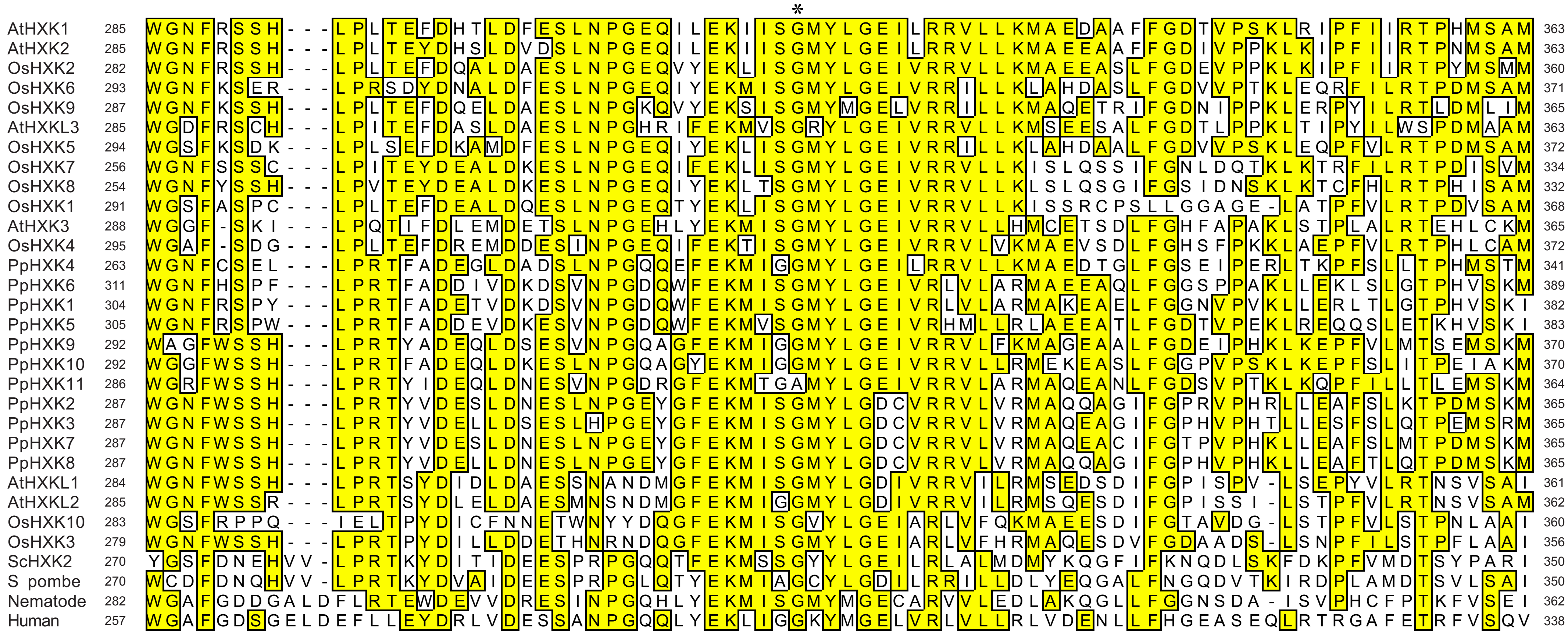
*
AtHXK1 285 WGNFRSSH---LPLTEFDHTLDFESLNPGEQILEKIISGMYLGEILRRVLLKMAEDAAFFGDTVPSKLRIPFIIRTPHMSAM 363
AtHXK2 285 WGNFRSSH---LPLTEYDHSLDVDSLNPGEQILEKIISGMYLGEILRRVLLKMAEEAAFFGDIVPPKLKIPFIIRTPNMSAM 363
OsHXK2 282 WGNFRSSH---LPLTEFDQALDAESLNPGEQVYEKLISGMYLGEIVRRVLLKMAEEASLFGDEVPPKLKIPFIIRTPYMSMM 360
OsHXK6 293 WGNFKSER---LPRSDYDNALDFESLNPGEQIYEKMISGMYLGEIVRRILLKLAHDASLFGDVVPTKLEQRFILRTPDMSAM 371
OsHXK9 287 WGNFKSSH---LPLTEFDQELDAESLNPGKQVYEKSISGMYMGELVRRILLKMAQETRIFGDNIPPKLERPYILRTLDMLIM 365
AtHXKL3 285 WGDFRSCH---LPITEFDASLDAESLNPGHRIFEKMVSGRYLGEIVRRVLLKMSEESALFGDTLPPKLTIPYILWSPDMAAM 363
OsHXK5 294 WGSFKSDK---LPLSEFDKAMDFESLNPGEQIYEKLISGMYLGEIVRRILLKLAHDAALFGDVVPSKLEQPFVLRTPDMSAM 372
OsHXK7 256 WGNFSSSC---LPITEYDEALDKESLNPGEQIFEKLISGMYLGEIVRRVLLKISLQSSIFGNLDQTKLKTRFILRTPDISVM 334
OsHXK8 254 WGNFYSSH---LPVTEYDEALDKESLNPGEQIYEKLTSGMYLGEIVRRVLLKLSLQSGIFGSIDNSKLKTCFHLRTPHISAM 332
OsHXK1 291 WGSFASPC---LPLTEFDEALDQESLNPGEQTYEKLISGMYLGEIVRRVLLKISSRCPSLLGGAGE-LATPFVLRTPDVSAM 368
AtHXK3 288 WGGF-SKI---LPQTIFDLEMDETSLNPGEHLYEKMISGMYLGEIVRRVLLHMCETSDLFGHFAPAKLSTPLALRTEHLCKM 365
OsHXK4 295 WGAF-SDG---LPLTEFDREMDDESINPGEQIFEKTISGMYLGEIVRRVLVKMAEVSDLFGHSFPKKLAEPFVLRTPHLCAM 372
PpHXK4 263 WGNFCSEL---LPRTFADEGLDADSLNPGQQEFEKMIGGMYLGEILRRVLLKMAEDTGLFGSEIPERLTKPFSLLTPHMSTM 341
PpHXK6 311 WGNFHSPF---LPRTFADDIVDKDSVNPGDQWFEKMISGMYLGEIVRLVLARMAEEAQLFGGSPPAKLLEKLSLGTPHVSKM 389
PpHXK1 304 WGNFRSPY---LPRTFADETVDKDSVNPGDQWFEKMISGMYLGEIVRLVLARMAKEAELFGGNVPVKLLERLTLGTPHVSKI 382
PpHXK5 305 WGNFRSPW---LPRTFADDEVDKESVNPGDQWFEKMVSGMYLGEIVRHMLLRLAEEATLFGDTVPEKLREQQSLETKHVSKI 383
PpHXK9 292 WAGFWSSH---LPRTYADEQLDSESVNPGQAGFEKMIGGMYLGEIVRRVLFKMAGEAALFGDEIPHKLKEPFVLMTSEMSKM 370
PpHXK10 292 WGGFWSSH---LPRTFADEQLDKESLNPGQAGYEKMIGGMYLGEIVRRVLLRMEKEASLFGGPVPSKLKEPFSLITPEIAKM 370
PpHXK11 286 WGRFWSSH---LPRTYIDEQLDNESVNPGDRGFEKMTGAMYLGEIVRRVLARMAQEANLFGDSVPTKLKQPFILLTLEMSKM 364
PpHXK2 287 WGNFWSSH---LPRTYVDESLDNESLNPGEYGFEKMISGMYLGDCVRRVLVRMAQQAGIFGPRVPHRLLEAFSLKTPDMSKM 365
PpHXK3 287 WGNFWSSH---LPRTYVDELLDSESLHPGEYGFEKMISGMYLGDCVRRVLVRMAQEAGIFGPHVPHTLLESFSLQTPEMSRM 365
PpHXK7 287 WGNFWSSH---LPRTYVDESLDNESLNPGEYGFEKMISGMYLGDCVRRVLVRMAQEACIFGTPVPHKLLEAFSLMTPDMSKM 365
PpHXK8 287 WGNFWSSH---LPRTYVDELLDNESLNPGEYGFEKMISGMYLGDCVRRVLVRMAQQAGIFGPHVPHKLLEAFTLQTPDMSKM 365
AtHXKL1 284 WGNFWSSH---LPRTSYDIDLDAESSNANDMGFEKMISGMYLGDIVRRVILRMSEDSDIFGPISPV-LSEPYVLRTNSVSAI 361
AtHXKL2 285 WGNFWSSR---LPRTSYDLELDAESMNSNDMGFEKMIGGMYLGDIVRRVILRMSQESDIFGPISSI-LSTPFVLRTNSVSAM 362
OsHXK10 283 WGSFRPPQ---IELTPYDICFNNETWNYYDQGFEKMISGVYLGEIARLVFQKMAEESDIFGTAVDG-LSTPFVLSTPNLAAI 360
OsHXK3 279 WGNFWSSH---LPRTPYDILLDDETHNRNDQGFEKMISGMYLGEIARLVFHRMAQESDVFGDAADS-LSNPFILSTPFLAAI 356
ScHXK2 270 YGSFDNEHVV-LPRTKYDITIDEESPRPGQQTFEKMSSGYYLGEILRLALMDMYKQGFIFKNQDLSKFDKPFVMDTSYPARI 350
S pombe 270 WCDFDNQHVV-LPRTKYDVAIDEESPRPGLQTYEKMIAGCYLGDILRRILLDLYEQGALFNGQDVTKIRDPLAMDTSVLSAI 350
Nematode 282 WGAFGDDGALDFLRTEWDEVVDRESINPGQHLYEKMISGMYMGECARVVLEDLAKQGLLFGGNSDA-ISVPHCFPTKFVSEI 362
Human 257 WGAFGDSGELDEFLLEYDRLVDESSANPGQQLYEKLIGGKYMGELVRLVLLRLVDENLLFHGEASEQLRTRGAFETRFVSQV 338

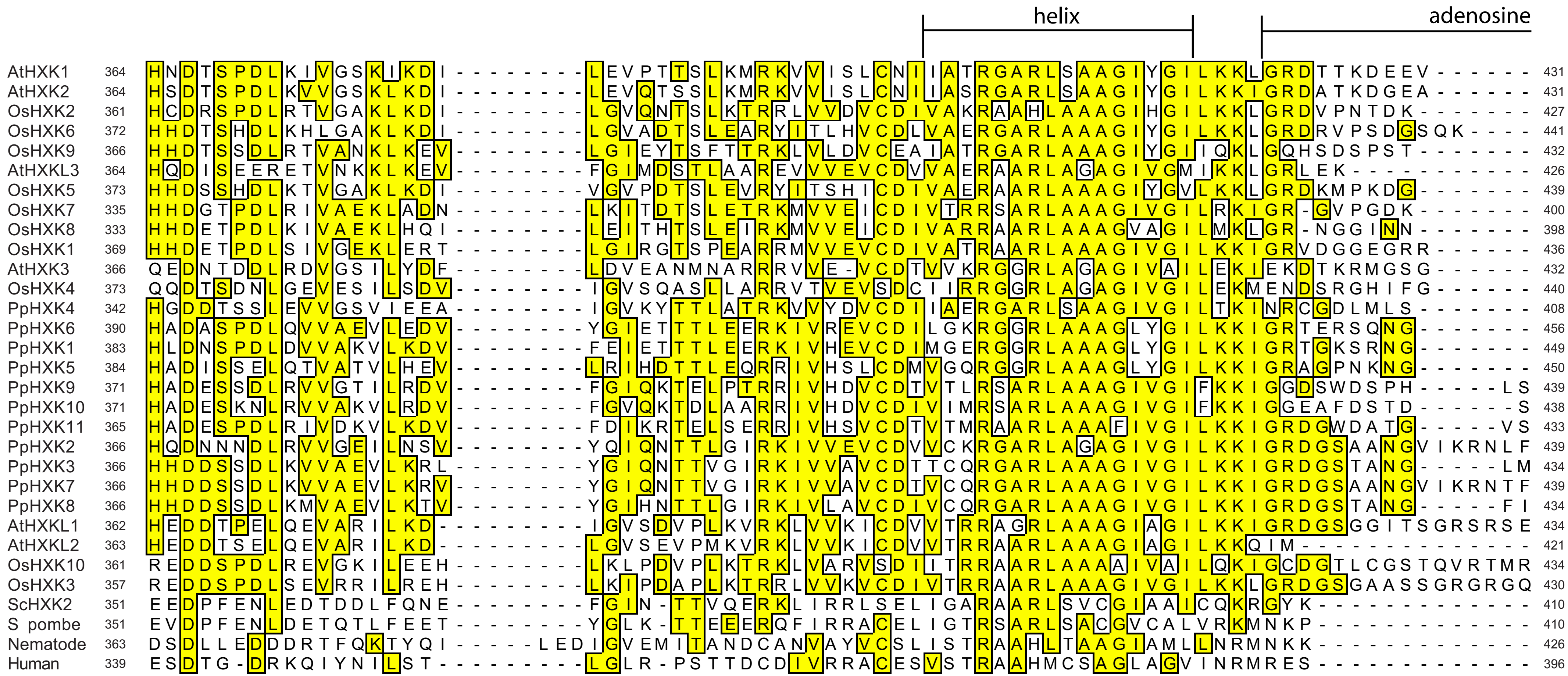
helix
adenosine
AtHXK1
AtHXK2
OsHXK2
OsHXK6
OsHXK9
AtHXKL3
OsHXK5
OsHXK7
OsHXK8
OsHXK1
AtHXK3
OsHXK4
PpHXK4
PpHXK6
PpHXK1
PpHXK5
PpHXK9
PpHXK10
PpHXK11
PpHXK2
PpHXK3
PpHXK7
PpHXK8
AtHXKL1
AtHXKL2
OsHXK10
OsHXK3
ScHXK2
S pombe
Nematode
Human

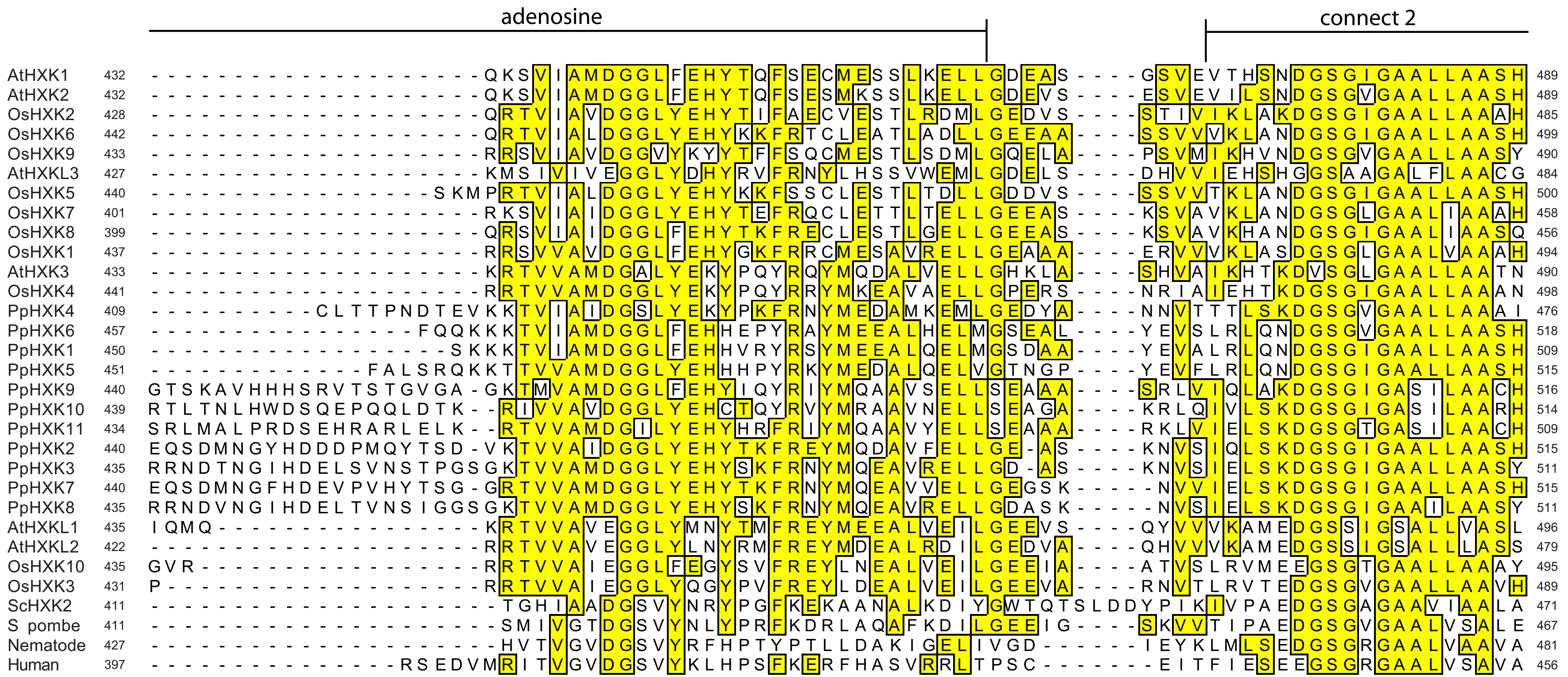
adenosine
connect 2
AtHXK1 432 --------------------QKSVIAMDGGLFEHYTQFSECMESSLKELLGDEAS----GSVEVTHSNDGSGIGAALLAASH 489
AtHXK2 432 --------------------QKSVIAMDGGLFEHYTQFSESMKSSLKELLGDEVS----ESVEVILSNDGSGVGAALLAASH 489
OsHXK2 428 --------------------QRTVIAVDGGLYEHYTIFAECVESTLRDMLGEDVS----STIVIKLAKDGSGIGAALLAAAH 485
OsHXK6 442 --------------------QRTVIALDGGLYEHYKKFRTCLEATLADLLGEEAA----SSVVVKLANDGSGIGAALLAASH 499
OsHXK9 433 --------------------RRSVIAVDGGVYKYYTFFSQCMESTLSDMLGQELA----PSVMIKHVNDGSGVGAALLAASY 490
AtHXKL3 427 --------------------KMSIVIVEGGLYDHYRVFRNYLHSSVWEMLGDELS----DHVVIEHSHGGSAAGALFLAACG 484
OsHXK5 440 -----------------SKMPRTVIALDGGLYEHYKKFSSCLESTLTDLLGDDVS----SSVVTKLANDGSGIGAALLAASH 500
OsHXK7 401 --------------------RKSVIAIDGGLYEHYTEFRQCLETTLTELLGEEAS----KSVAVKLANDGSGLGAALIAAAH 458
OsHXK8 399 --------------------QRSVIAIDGGLFEHYTKFRECLESTLGELLGEEAS----KSVAVKHANDGSGIGAALIAASQ 456
OsHXK1 437 --------------------RRSVVAVDGGLFEHYGKFRRCMESAVRELLGEAAA----ERVVVKLASDGSGLGAALVAAAH 494
AtHXK3 433 --------------------KRTVVAMDGALYEKYPQYRQYMQDALVELLGHKLA----SHVAIKHTKDVSGLGAALLAATN 490
OsHXK4 441 --------------------RRTVVAMDGGLYEKYPQYRRYMKEAVAELLGPERS----NRIAIEHTKDGSGIGAALLAAAN 498
PpHXK4 409 ----------CLTTPNDTEVKKTVIAIDGSLYEKYPKFRNYMEDAMKEMLGEDYA---NNVTTTLSKDGSGVGAALLAAAI 476
PpHXK6 457 ----------------FQQKKKTVIAMDGGLFEHHEPYRAYMEEALHELMGSEAL---YEVSLRLQNDGSGVGAALLAASH 518
PpHXK1 450 ------------------SKKKTVIAMDGGLFEHHVRYRSYMEEALQELMGSDAA---YEVALRLQNDGSGIGAALLAASH 509
PpHXK5 451 --------------FALSRQKKTVVAMDGGLYEHHHPYRKYMEDALQELVGTNGP---YEVFLRLQNDGSGIGAALLAASH 515
PpHXK9 440 GTSKAVHHHSRVTSTGVGA-GKTMVAMDGGLFEHYIQYRIYMQAAVSELLSEAAA---SRLVIQLAKDGSGIGASILAACH 516
PpHXK10 439 RTLTNLHWDSQEPQQLDTK--RIVVAVDGGLYEHCTQYRVYMRAAVNELLSEAGA---KRLQIVLSKDGSGIGASILAARH 514
PpHXK11 434 SRLMALPRDSEHRARLELK--RTVVAMDGILYEHYHRFRIYMQAAVYELLSEAAA---RKLVIELSKDGSGTGASILAACH 509
PpHXK2 440 EQSDMNGYHDDDPMQYTSD-VKTVVAIDGGLYEHYTKFREYMQDAVFELLGE-AS---KNVSIQLSKDGSGIGAALLAASH 515
PpHXK3 435 RRNDTNGIHDELSVNSTPGSGKTVVAMDGGLYEHYSKFRNYMQEAVRELLGD-AS---KNVSIELSKDGSGIGAALLAASY 511
PpHXK7 440 EQSDMNGFHDEVPVHYTSG-GRTVVAMDGGLYEHYTKFRNYMQEAVVELLGEGSK----NVVIELSKDGSGIGAALLAASH 515
PpHXK8 435 RRNDVNGIHDELTVNSIGGSGKTVVAMDGGLYEHYSKFRNYMQEAVRELLGDASK----NVSIELSKDGSGIGAAILAASY 511
AtHXKL1 435 IQMQ----------------KRTVVAVEGGLYMNYTMFREYMEEALVEILGEEVS---QYVVVKAMEDGSSIGSALLVASL 496
AtHXKL2 422 --------------------RRTVVAVEGGLYLNYRMFREYMDEALRDILGEDVA---QHVVVKAMEDGSSIGSALLLASS 479
OsHXK10 435 GVR-----------------RRTVVAIEGGLFEGYSVFREYLNEALVEILGEEIA---ATVSLRVMEEGSGTGAALLAAAY 495
OsHXK3 431 P-------------------RRTVVAIEGGLYQGYPVFREYLDEALVEILGEEVA---RNVTLRVTEDGSGVGAALLAAVH 489
ScHXK2 411 ---------------------TGHIAADGSVYNRYPGFKEKAANALKDIYGWTQTSLDDYPIKIVPAEDGSGAGAAVIAALA 471
S pombe 411 ---------------------SMIVGTDGSVYNLYPRFKDRLAQAFKDILGEEIG----SKVVTIPAEDGSGVGAALVSALE 467
Nematode 427 ---------------------HVTVGVDGSVYRFHPTYPTLLDAKIGELIVGD------IEYKLMLSEDGSGRGAALVAAVA 481
Human 397 ---------------RSEDVMRITVGVDGSVYKLHPSFKERFHASVRRLTPSC--------EITFIESEEGSGRGAALVSAVA 456

AtHXK1	490	S L Y L E D S	496
AtHXK2	490	S Q Y L E L E D D S E T S	502
OsHXK2	486	S Q Y R E A E E L	494
OsHXK6	500	S Q Y A S V E	506
OsHXK9	491	S Q Y H Q A E S A D S S	502
AtHXKL3	485	D G H Q D S E S K	493
OsHXK5	501	S Q Y A E I D	507
OsHXK7	459	S Q Y L N	463
OsHXK8	457	S R	458
OsHXK1	495	S Q R A	498
AtHXK3	491	S I Y	493
OsHXK4	499	S K Y A A A Q I S T R	509
PpHXK4	477	S T E L R L G D V G V T K	489
PpHXK6	519	S Q F K	522
PpHXK1	510	S H F K	513
PpHXK5	516	S R H R Q T E	522
PpHXK9	517	S K Y R	520
PpHXK10	515	S Q H R	518
PpHXK11	510	S E F A P S Y S	517
PpHXK2	516	A E H L S S	521
PpHXK3	512	A E Y V P S	517
PpHXK7	516	A E Y V I S	521
PpHXK8	512	A E Y V P S	517
AtHXKL1	497	Q S	498
AtHXKL2	480	Q S V Q T I P S V	488
OsHXK10	496	S S A R Q K N S E	504
OsHXK3	490	S S N R Q Q Q G G P I	500
ScHXK2	472	Q K R I A E G K S V G I I G A	486
S pombe	468	A K G K A L T S D I L A E H L K N	484
Nematode	482	T R L K E E K L A A S L S S S S S N Q	500
Human	457	C K K A C M L G Q	465
