# Supplementary figures and images for: Two novel types of hexokinases in the moss Physcomitrella patens
Source: BMC Plant Biol. 2011 Feb 14;11:32. doi: 10.1186/1471-2229-11-32 (PMC3045890; doi:10.1186/1471-2229-11-32)

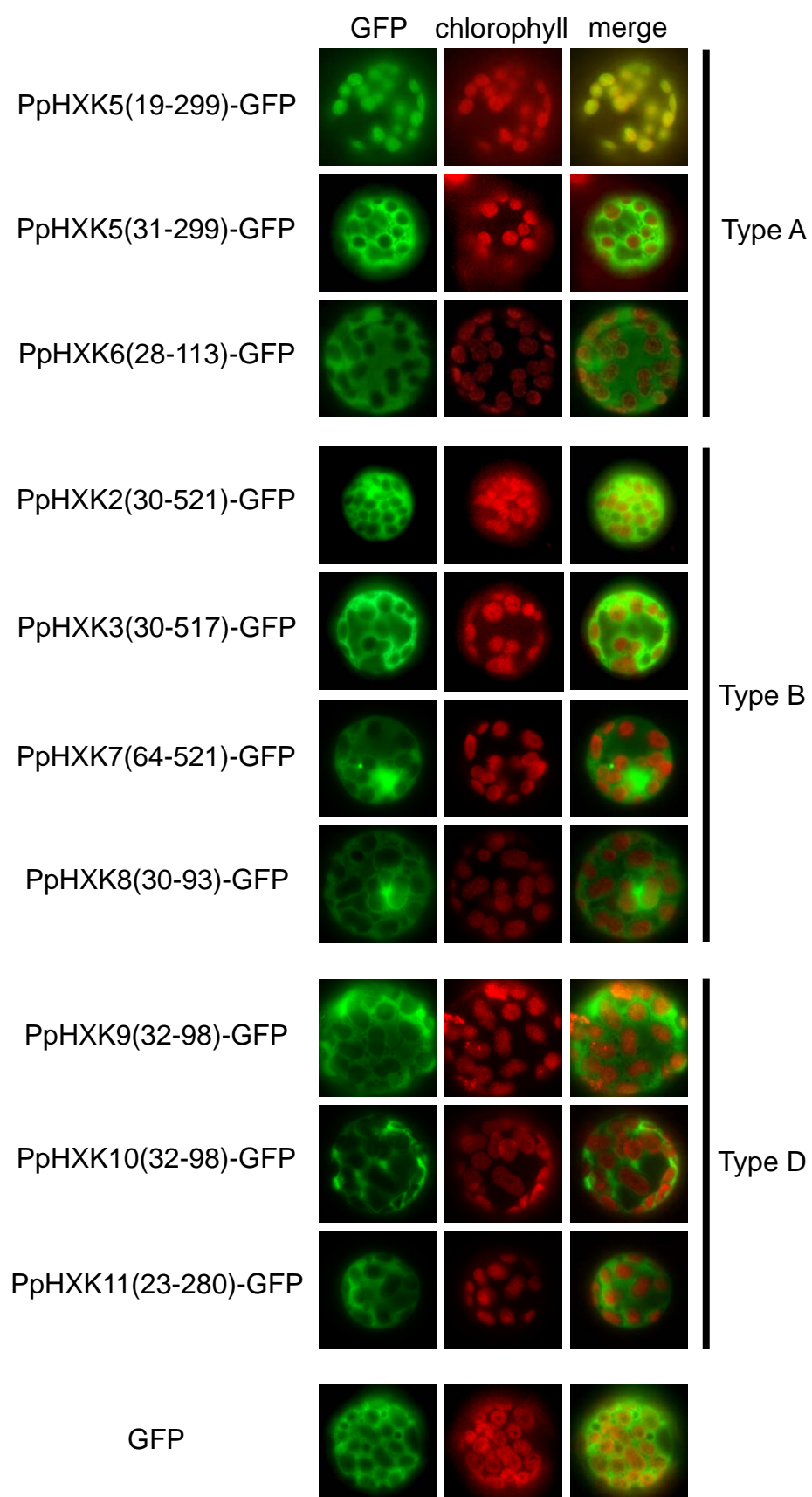

Supplement: Additional file 6 — Intracellular localization of truncated hexokinase-GFP fusions. Fluorescence microscopy pictures of wild type moss protoplasts transiently expressing different truncated versions of the Physcomitrella hexokinases fused to GFP. The hexokinase codons that were fused in frame to GFP are indicated for each hexokinase. GFP fluorescence is shown in green, with chlorophyll auto-fluorescence in red serving as a chloroplast marker. Protoplasts expressing GFP alone were also included as a control. [file 1471-2229-11-32-S6.PDF]
