# Supplementary material for: Two novel types of hexokinases in the moss Physcomitrella patens
Source: BMC Plant Biol. 2011 Feb 14;11:32. doi: 10.1186/1471-2229-11-32 (PMC3045890; doi:10.1186/1471-2229-11-32)
Supplement: Additional file 7 — Sequence polymorphism in the PpHXK3 promoter and alternative splicing of the PpHXK7 transcript. a. Microsatellite repeat in the PpHXK3 promoter that shows evidence of rapid evolution. The two sequence variants are shown. b. The two splice variants of PpHXK7 with (PpHXK7b) and without (PpHXK7a) an N-terminal membrane anchor. The nucleotide sequences and predicted encoded peptide sequences of the two splice variants are shown. The splice sites and the start codons are underlined, and the methionines are highlighted by a black background. [file 1471-2229-11-32-S7.PDF]

a

```
PpHXK3a  CGCGCTGCAGAGTCCGGGTTAGCGAGAGAGAGAGAGAGAGCGAGAGAGCTGGAAGCGAGA
PpHXK3b  CGCGCTGCAGAGTCCGGGTTAGCGAGAGAGAGAGAGAG--CGAGAGAGCTGGAAGCGAGA
```

b

```
PpHXK7a  CGAGACTTTTTAGAAGAG------------------------------------------
PpHXK7b  CGAGACTTTTTAGAAGAGGTGAGGAGGCGAAGAAAGTGAGCTTTTATTTTTATTTGTGTG

PpHXK7a  ------------------------------------------------------------
PpHXK7b  TGTGTGAGAGAGAGGGAGGGGTAGAGACAGAGGAGAGGAAAAATGACACAATCGAAGGTA
                                                    M  T  Q  S  K  V

PpHXK7a  ----------------------------CTGCGGCGTGCGCTGCTGCGGCTGTGATTGTA
PpHXK7b  ATGACGGGCGTGTACATCGCCTGCGCAGCTGCGGCGTGCGCTGCTGCGGCTGTGATTGTA
          M  T  G  V  Y  I  A  C  A  A  A  A  C  A  A  A  A  V  I  V

PpHXK7a  TCACGGCGCTTGAAGGTTCGATCACAGAAATGCACTGCGCGGAAAATTCTGCTGGAGTTT
PpHXK7b  TCACGGCGCTTGAAGGTTCGATCACAGAAATGCACTGCGCGGAAAATTCTGCTGGAGTTT
          S  R  R  L  K  V  R  S  Q  K  C  T  A  R  K  I  L  L  E  F

PpHXK7a  CAGGAGGCCTGTTACACGCCTTTGGCGCGCCTGCGCCAGGTGGTGGATGCTATGGCGGTC
PpHXK7b  CAGGAGGCCTGTTACACGCCTTTGGCGCGCCTGCGCCAGGTGGTGGATGCTATGGCGGTC
          Q  E  A  C  Y  T  P  L  A  R  L  R  Q  V  V  D  A  M  A  V
```
