# Supplementary material for: Two novel types of hexokinases in the moss Physcomitrella patens
Source: BMC Plant Biol. 2011 Feb 14;11:32. doi: 10.1186/1471-2229-11-32 (PMC3045890; doi:10.1186/1471-2229-11-32)
Supplement: Additional file 8 — Alternative splicing of Physcomitrella hexokinase transcripts. The different Physcomitrella hexokinase transcripts showing alternative splicing. The plasmids names and the type of alternative splicing within these transcripts are listed together with the predicted effect on the expressed protein. [file 1471-2229-11-32-S8.PDF]

TABLE S5

Alternative splicing of Physcomitrella hexokinase transcripts

Gene	Plasmid	Type of alternative splicing	Effect on predicted protein sequence
PpHXK3	pTO30	Alternative 5’ donor site in exon 2	Premature termination
PpHXK5	pdp18063	Retention of intron 6<br>Retention of intron 7	Premature termination
PpHXK5	pdp33748	Retention of intron 3<br>Alternative 5’ donor site in intron 4<br>Retention of intron 5<br>Retention of intron 7	Premature termination
PpHXK7	pdp03464	Splicing between 5’-UTR and exon 1	No membrane anchor
PpHXK9	pAN24	Retention of intron 1<br>Alternative 5’ donor site in exon 5	Premature termination
PpHXK10	pAN30	Alternative 5’ donor site in intron 1<br>Alternative 5’ donor site in intron 2<br>Alternative 3’ acceptor site in intron 2<br>Retantion of intron 3	Premature termination
PpHXK10	pAN31	Alternative 5’ donor site in intron 1<br>Retention of intron 3<br>Alternative 5’ donor site in exon 5<br>Alternative 3’ acceptor site in exon 9	Premature termination
PpHXK10	pAN32	Alternative 5’ donor site in intron 2<br>Retention of intron 3<br>Alternative 3’ acceptor site in intron 4<br>Alternative 5’ donor site in exon 5<br>Alternative 3’ acceptor site in exon 8	Premature termination
PpHXK11	pAN23	Alternative 5’ donor site in exon 5<br>Skipping of exon 8	Frame shift and premature termination
