# Supplementary material for: Two novel types of hexokinases in the moss Physcomitrella patens
Source: BMC Plant Biol. 2011 Feb 14;11:32. doi: 10.1186/1471-2229-11-32 (PMC3045890; doi:10.1186/1471-2229-11-32)
Supplement: Additional file 9 — Accession numbers for Physcomitrella hexokinases. The accession numbers of the PpHXK2-pPHXK11 transcripts and the corresponding GeneIDs are listed. [file 1471-2229-11-32-S9.PDF]

TABLE S6

Accession numbers for *Physcomitrella* hexokinases

Gene	mRNA	GeneID
PpHXK2	XM_001784578.1	5947828
PpHXK3	XM_001784282.1	5947550
PpHXK4	XM_001760896.1	5924067
PpHXK5	XM_001766381.1	5929648
PpHXK6	XM_001762899.1	5926130
PpHXK7	XM_001754096.1	5917393
PpHXK8	XM_001752177.1	5915312
PpHXK9	XM_001770125.1	5933371
PpHXK10	XM_001776713.1	5939952
PpHXK11	XM_001779426.1	5942660
